# Quantifying the hydroxyapatite orientation near the ossification front in a piglet femoral condyle using X-ray diffraction tensor tomography

**DOI:** 10.1038/s41598-020-80615-4

**Published:** 2021-01-25

**Authors:** Fredrik K. Mürer, Basab Chattopadhyay, Aldritt Scaria Madathiparambil, Kim Robert Tekseth, Marco Di Michiel, Marianne Liebi, Magnus B. Lilledahl, Kristin Olstad, Dag W. Breiby

**Affiliations:** 1 PoreLab, Department of Physics, Norwegian University of Science and Technology (NTNU), Høgskoleringen 5, 7491 Trondheim, Norway; 2 ESRF-The European Synchrotron, 71 Avenue des Martyrs, 38000 Grenoble, France; 3 Chalmers University of Technology, 412 96 Gothenburg, Sweden; 4 Department of Physics, Norwegian University of Science and Technology (NTNU), Høgskoleringen 5, 7491 Trondheim, Norway; 5 Faculty of Veterinary Medicine, Department of Companion Animal Clinical Sciences, Norwegian University of Life Sciences (NMBU), Equine Section, Sentrum, P. O. Box 369, 0102 Oslo, Norway; 6 Department of Microsystems, University of South-Eastern Norway (USN), Campus Vestfold, 3184 Borre, Norway

**Keywords:** Nanoscale biophysics, Biomineralization, Imaging techniques

## Abstract

While a detailed knowledge of the hierarchical structure and morphology of the extracellular matrix is considered crucial for understanding the physiological and mechanical properties of bone and cartilage, the orientation of collagen fibres and carbonated hydroxyapatite (HA) crystallites remains a debated topic. Conventional microscopy techniques for orientational imaging require destructive sample sectioning, which both precludes further studies of the intact sample and potentially changes the microstructure. In this work, we use X-ray diffraction tensor tomography to image non-destructively in 3D the HA orientation in a medial femoral condyle of a piglet. By exploiting the anisotropic HA diffraction signal, 3D maps showing systematic local variations of the HA crystallite orientation in the growing subchondral bone and in the adjacent mineralized growth cartilage are obtained. Orientation maps of HA crystallites over a large field of view (~ 3 × 3 × 3 mm^3^) close to the ossification (bone-growth) front are compared with high-resolution X-ray propagation phase-contrast computed tomography images. The HA crystallites are found to predominantly orient with their crystallite *c*-axis directed towards the ossification front. Distinct patterns of HA preferred orientation are found in the vicinity of cartilage canals protruding from the subchondral bone. The demonstrated ability of retrieving 3D orientation maps of bone-cartilage structures is expected to give a better understanding of the physiological properties of bones, including their propensity for bone-cartilage diseases.

## Introduction

Understanding the microstructure of subchondral bone and growth cartilage is important, also for the development of new synthetic implants^1^ and for curing bone and cartilage diseases with high socioeconomic impact, such as osteochondrosis^2^ and osteoarthritis^3^. Lengthwise bone growth in young animals is a complex process, which includes deposition of bone mineral on a collagen matrix and the incorporation of vasculature from the growth cartilage into the newly formed subchondral bone^2^. Additionally, the mechanical properties of bone depend on the *orientation* of both mineral and collagen fibrils^4^. X-ray computed tomography (CT) has been widely used both for bone and cartilage research and diagnostics since the 1970s, but conventional CT yields only scalar fields describing the material density. Consequently, there is a need for non-destructive 3D imaging techniques capable of imaging bone and cartilage with both chemical and orientational contrast with high resolution across a wide field of view.

Subchondral bone is found close to the bone-cartilage interfaces at the proximal and distal ends of long bones^5^. The immature subchondral bone can be classified into primary and secondary spongiosa, depending on the distance from the bone-cartilage interfaces and the morphology of the trabecula^6^. The primary spongiosa is the newly formed bone close to the bone-cartilage interface, while the secondary spongiosa is found deeper into the bone. At the microscale, the trabeculae are composed of mineralized collagen I fibrils, organized into lamellae^7^. The mineralized phase consists of carbonated hydroxyapatite (HA) crystallites, providing the stiffness of bone^8^. Carbonated HA crystallites are located within the collagen fibril gap zones and outside the fibrils^7^. The HA crystallites are generally described as thin highly anisotropic crystals^9,10^, with the HA hexagonal unit cell *c*-axis usually assumed to coincide with the crystallite long axis exhibiting preferred orientation along the collagen fibril axis^11–13^. Additionally, the HA crystallites are reported to have rotational symmetry about the collagen fibril axis^13–15^, a claim which has also recently come under renewed scrutiny^16^. The ends of long bones are covered by articular cartilage, and in immature individuals, there is a layer of growth cartilage between the articular cartilage and the subchondral bone. Articular cartilage and growth cartilage are composed mainly of collagen II fibrils, proteoglycans, chondrocytes in cartilage matrix cavities (lacunae), and 65–80 wt.% water^17^. Growth cartilage, as opposed to articular cartilage, contains cartilage canals^2^, which are tubular structures supplying mesenchymal cells, oxygen and nutrition through internal arterioles and venules. Collapse of cartilage canals close to the bone-cartilage ossification front, perhaps related to variations in the mineralized extracellular matrix, has been shown to be associated with osteochondrosis in pigs^18^.

Collagen fibril orientation can be mapped out for physically cut 2D sections by optical techniques utilizing the polarization of light, easily available in conventional light microscopy^19^, Raman microscopy^20^, or second harmonic generation microscopy^21^. Most of the known models of collagen orientation, e.g. the twisted plywood model^22^ and distribution of HA crystallites in the collagen matrix, are based on transmission electron microscopy, and recent advances include imaging of single HA crystallites^7^. Several important studies of bone have also been made based on scanning electron microscopy in combination with focused ion beam milling, revealing previously unknown hierarchical levels and a weakly ordered minor component with randomly oriented individual collagen fibrils, with crystals inside and possibly between fibrils^23,24^. However, the optical and electron microscopy techniques require destructive sample processing, which is non-trivial for bone because of the calcified matrix. The two basic preparation methods for histology are (i) decalcification by acid solutions to study the cells and the organic matrix, and (ii) grinding of dried bone to study the details of the calcified matrix—both obviously giving massive modifications to the sample under study^25,26^. In the case of cartilage, drying may significantly change the morphology^27^, discouraging the use of vacuum dependent techniques.

Conventional attenuation and phase-contrast based X-ray imaging methods have several widely known advantages for bone research, notably high penetration and excellent contrast, allowing the samples to be measured in their natural hydrated state with minimal sample damage^28^. However, conventional X-ray imaging is strongly limited when it comes to distinguishing materials of similar electron density^28^. Small-angle X-ray scattering (SAXS) and wide-angle X-ray diffraction (XRD) are routinely used in the material sciences to resolve local structures and to measure texture in anisotropic samples^29^, including bone structures^13,30^ and their orientation distributions^13^. SAXS and XRD signals can be combined with CT analysis (SAXS-CT; XRD-CT), facilitating 3D imaging with material composition or crystallite unit cell parameters as contrast mechanism, thus allowing materials of similar density to be distinguished^31–35^. The spatially resolved preferred orientation of well-defined scattering objects within the specimen can also be extracted using SAXS-CT and XRD-CT, first demonstrated on simple nanostructure orientations^33,36–38^ and later developed for retrieving more complex crystallite orientation arrangements in six-dimensional SAXS-CT^39^ and small-angle X-ray scattering tensor tomography (SASTT)^40, 41^. In SASTT, many (~ 10^6^) X-ray diffraction patterns are collected and used to reconstruct quantitative 3D images depicting the spatial distribution of specific material compounds and/or their orientation within the sample. Most recently, X-ray diffraction tensor tomography (XRDTT), which is the wide-angle analogue to SASTT, was used to demonstrate systematic localized orientational variations in human lamellar bone on a length scale of about 5 μm^16^.

Here, we demonstrate wide field-of-view (FoV ~ (3 mm)^3^) XRDTT as applied to the immature cartilage-bone interface in two femorotibial samples from a young piglet. The wide FoV enabled us to investigate the systematic variation of bone mineral HA in the newly developed bone close to the bone-cartilage interface (ossification front), as well as studying local HA orientation variations around cartilage canals protruding into the ossifying bone. The article is organized as follows. First, we present the ability of discriminating the different materials constituting the specimen, specifically, bone, cartilage, the immersing ethanol–water solution, and polyimide sample container by using the measured scattering to reconstruct material-specific 3D tomograms. In this work we refer to this approach as XRD-CT, in accordance with the first demonstrations of the technique^31^. Second, based on the anisotropy of the XRD and SAXS signals, we retrieve 3D maps of the HA crystallite orientation distribution function throughout the bone/cartilage specimens, which are presented together with complementary high-resolution propagation phase-contrast X-ray CT (PPC-CT) data. This information is clearly unique, and potentially of great importance for understanding the physiological properties of the ossifying bone, including its propensity to a wide range of diseases.

## Results

### Morphology and material specific maps of the sample interior obtained by phase and diffraction contrast tomography

**Figure 1:**
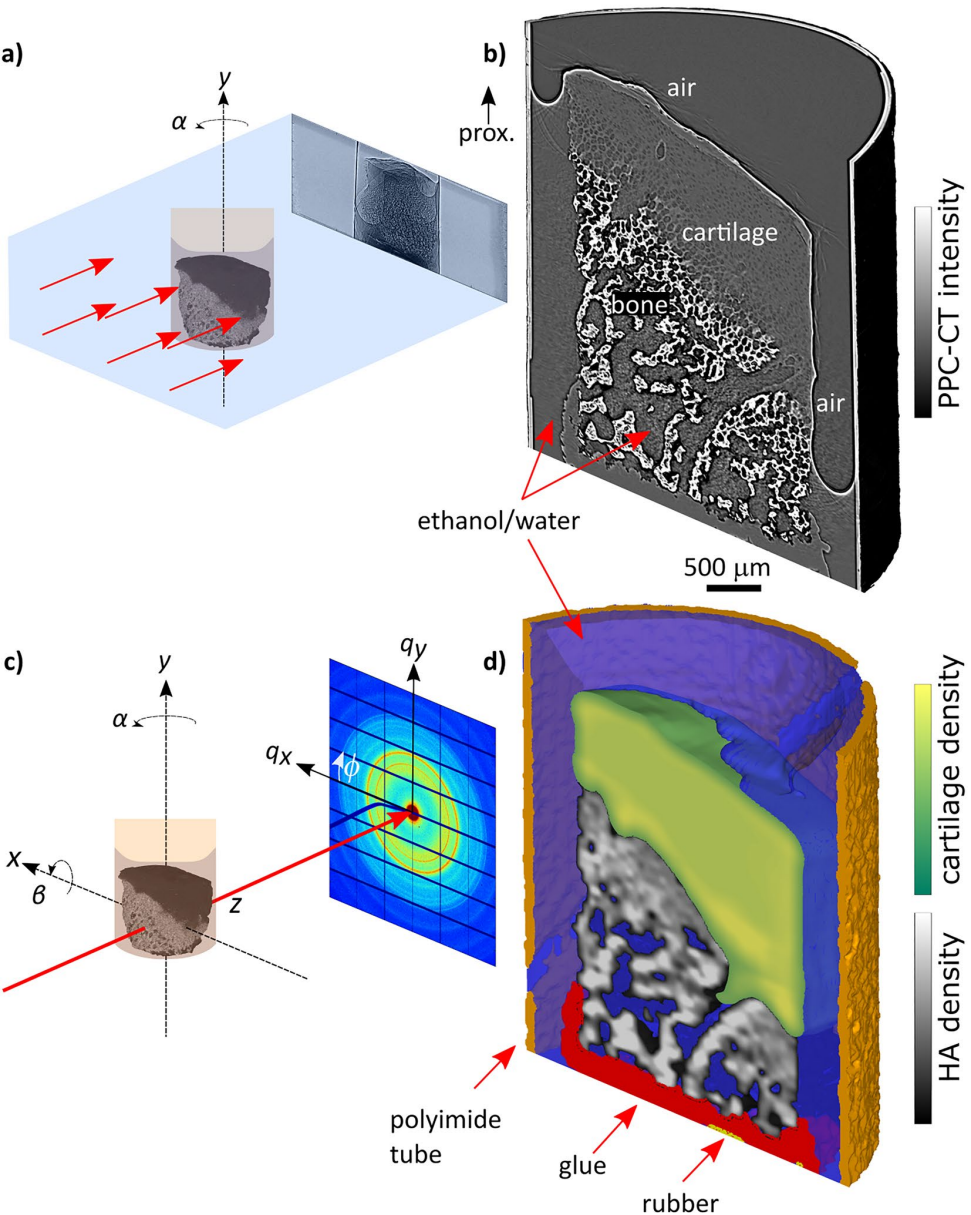
**(a)** Sketch of PPC-CT experimental setup. The sample is illuminated by a full-field X-ray beam, while rotated around *y.* Edge-enhanced propagation phase-contrast projections are recorded on the detector if the sample-detector distance is sufficiently long. **(b)** PPC-CT cutaway view of a bone/cartilage sample including the ethanol–water solution and polyimide sample container. *Prox.* proximal. **(c)** Sketch of a generic SASTT or XRDTT experimental setup. The sample is mounted onto a goniometer stage, allowing the sample to be raster-scanned in (*x*,*y*) and rotated around *y* (angle *α*) and *x* (angle *β*). A collimated X-ray beam penetrates the sample and 2D diffraction patterns are collected on the detector. **(d)** Material specific XRD-CT, cutaway view.

Using propagation phase-contrast CT (PPC-CT) and X-ray diffraction CT (XRD-CT) we obtained tomograms with a voxel size of 3.2 µm and 50 μm, respectively, cf. Figure 1, which also provides principle sketches of the two setups. Cutaway views of a sample including the polyimide container are shown in Fig. 1b,d. The complementarity of the two X-ray microscopy techniques is immediately appreciated: while PPC-CT excels in resolution, XRD-CT allows discriminating the materials based on the diffraction signal. For PPC-CT, as for conventional attenuation-based CT, the strongly absorbing bone provides high contrast. Evidently, PPC-CT gives high-quality 3D images also of the weakly absorbing cartilage^42^ without the need of any contrast agent, because the sensitivity is increased by X-ray refraction in addition to absorption^28^. For the material-specific compound XRD-CT tomogram shown in Fig. 1d, the various materials were determined in separate reconstruction steps, cf. Materials and Methods. The presence of bone and mineralized cartilage was based on the integrated intensity of the HA002 peak, while cartilage was detected by small-angle scattering from collagen fibrils^43^.

**Figure 2:**
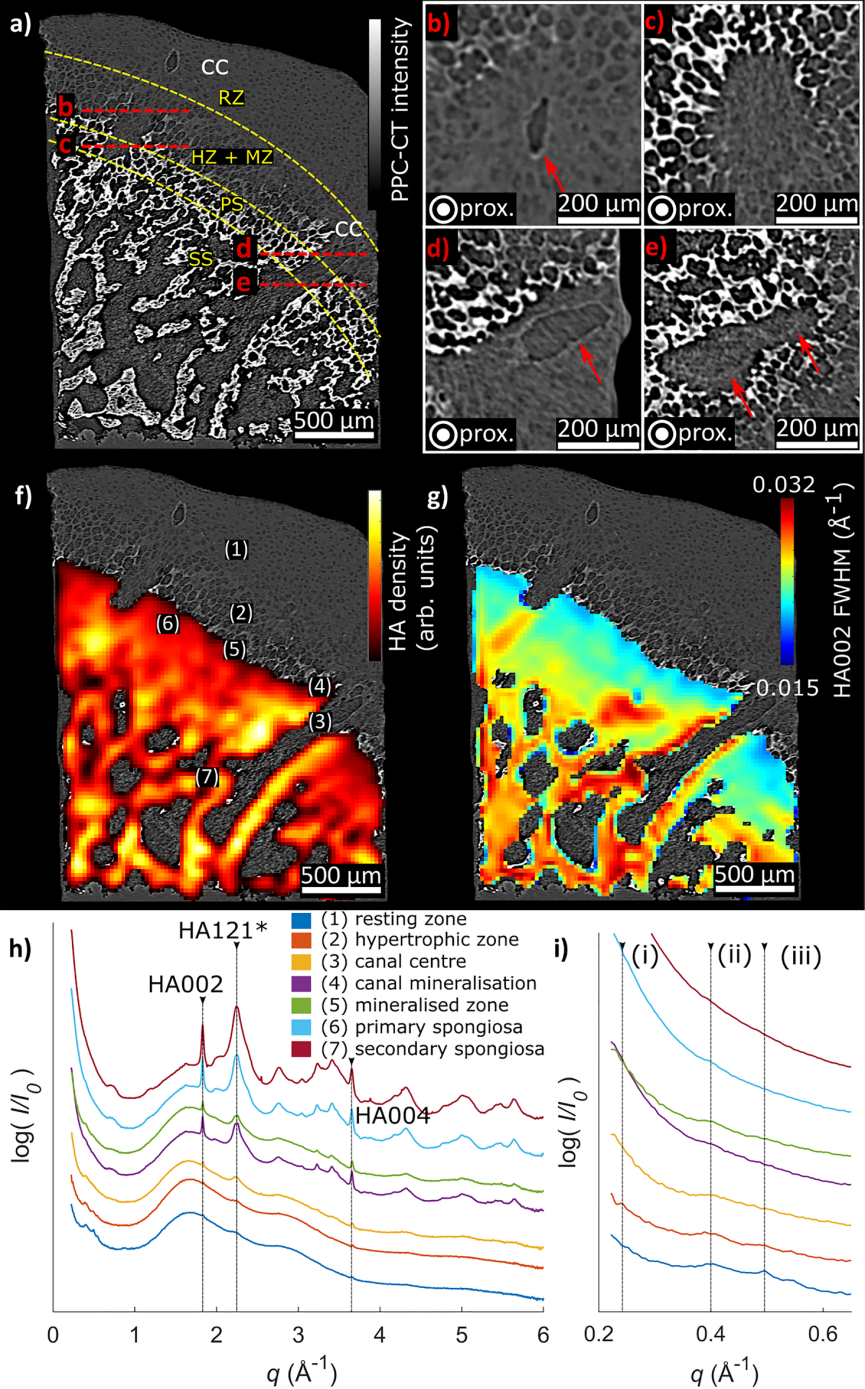
Cross-sectional view of the sample, showing the bone morphology in a region where two cartilage canals (cc) protrude into the bone. **(a)** 2D PPC-CT cross-section, with distinct zones in cartilage and bone delineated with yellow dashed curves. The red dashed lines give the location of the orthogonal (out of plane) magnified sections shown in **(b–e)**. *RZ* resting zone, *HZ* hypertrophic zone, *MZ* mineralized zone, *PS* primary spongiosa, *SS* secondary spongiosa, *cc* cartilage canal, *prox.* proximal. **(b–e)** Magnified out-of-plane oriented cross-sections corresponding to the red dashed lines in **(a)**. The red arrows indicate cartilage canal walls. **(f)** XRD-CT cross-section of the local HA density, overlaid with the PPC-CT cross-section from **(a)**. **(g)** Spatial variations of the HA002 peak width, showing that the peak width decreases towards the ossification front. The XRD-CT cross-sections in **(f,g)** have been up sampled by a factor 2 for display purposes. **(h)** XRD-CT reconstructed 1D diffractograms for the regions marked with (1)–(7) in **(f)**, demonstrating that HA is observed in all mineralized regions. Small Bragg peaks are observed in the resting zone and hypertrophic zone of the cartilage around *q* = 0.5 Å^−1^*.* An intensity offset has been added for display purposes. (**i**) Magnified view of the low *q* scattering used for SAXS analysis. Weak collagen SAXS peaks indicated with (i, ii, iii) are associated with collagen interfibrillar spacings.

The cross-sectional views provided in Fig. 2 further demonstrate the complementary information from PPC-CT and XRD-CT. With PPC-CT, the weakly absorbing microstructural details of the growth cartilage, such as chondrocyte-containing lacunae and cartilage canals, are clearly visible due to refractive index differences^42,44^. Based on the cell shape and cell-to-matrix volume ratio, we classified the cartilage into different zones using the nomenclature established in conventional histology^45,46^, see annotations in Fig. 2a. A pronounced change in cartilage morphology is seen in the hypertrophic and mineralized zones (cf. Figure 2a), where the chondrocyte lacunae appear as circle-shaped low-density regions in the cartilage matrix, in agreement with Ref. ^46^. The primary and secondary spongiosa were distinguished based on the morphology of the trabeculae^6^. XRD-CT provided material specific 3D maps of the sample interior, with spatially resolved diffractograms as exemplified in Fig. 2h. Because the diffraction patterns for the studied bone and cartilage samples were *weakly* textured (cf. SI, Figure S2.1), we could first analyse the sample mineral composition and spatial crystal structure variations under the approximate assumption of isotropic scattering, using a reconstruction procedure based on the filtered backprojection (FBP) algorithm^47^. The XRD-CT reconstructions gave spatially resolved material specific maps for the full sample 3D volume, in the form of diffraction patterns associated with each voxel within the sample. The main diffraction peak HA002 was identified and fitted using a nonlinear fitting routine to provide maps of the radially integrated peak intensity and peak width (Δ*q*), as shown in Fig. 2f,g. In the superposed view of the PPC-CT and XRD-CT cross-sections given in Fig. 2f, close correspondence is seen between the dense (bright; highly attenuating and scattering) regions of the PPC-CT cross-section and the regions of pronounced HA002 diffracted intensity observed by XRD-CT.

As shown, maps of the local density of HA based on the radially integrated intensity of the HA002 diffraction peak were obtained. Albeit Rietveld refinement has previously been reported for bone^48^ and HA phantom samples^49,50^, in the current study we limited the analysis to fitting single isolated peaks. Only the HA002 peak (and HA004 peak, results not shown) could be reliably fitted for quantitative analysis because the other peaks were highly overlapping. The feature marked as HA121* in Fig. 2h in fact contains overlapping HA Bragg peaks, specifically HA121, 112, 202, and 300. However, as the reconstruction procedure assigns one full diffractogram to each voxel in the reconstructed specimen, we could also obtain the local variations of the HA002 peak *width* (FWHM), which is often associated with crystallite size and strain^51^. We observed that the HA002 peak width (Δ*q*) (cf. Figure 2g) varied between regions of mineralized cartilage, primary spongiosa and secondary spongiosa with values ranging from 0.015 to 0.032 Å^−1^ (FWHM), see also the Discussion. Similarly, for the HA002 peak *position* (*q*), which is related to the unit cell dimensions^22^, no variations were observed, indicating that the HA is of similar composition throughout the sample.

Diffractograms for selected sample regions are shown in Fig. 2h. The diffraction signal from the cartilage regions was dominated by scattering from the ethanol and water solution, seen as the broad peak in the diffractograms in the range 1–4 Å^−1^. The cartilage regions additionally exhibited small-angle scattering from collagen fibrils^52^ and weak Bragg peaks at *q* = 0.24, 0.40 and 0.49 Å^−1^ could be observed in the resting and hypertrophic zones (cf. Fig. 2h,i), likely originating from collagen in the extracellular matrix^53,54^. In the bone and mineralized cartilage regions, including the mineralized regions extending into the growth cartilage surrounding cartilage canals, the Bragg peaks were identified as HA^55^, cf. SI, Figure S2.2. Small-angle scattering was detected from both bone and cartilage (cf. Fig. 2h). The small-angle scattering from mineralized cartilage and bone was notably stronger than from the un-mineralized cartilage^43^, which can be ascribed to the larger electron density difference between the HA crystallites and the collagen.

### 3D maps of HA orientation in bone and mineralized cartilage obtained by XRDTT

**Figure 3:**
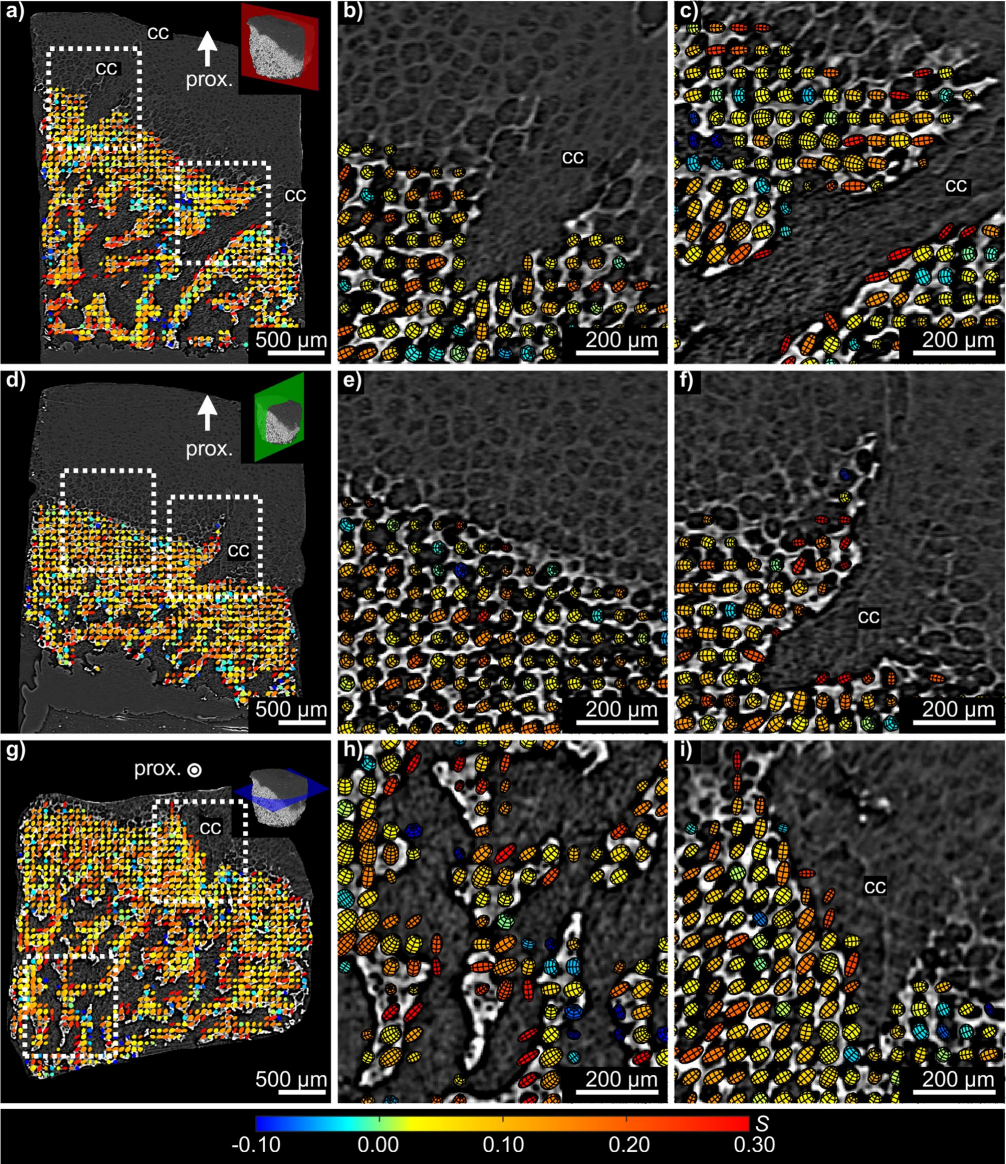
Registered cross-sections from XRDTT and PPC-CT. Variations in the preferred crystallite orientation of HA are shown for three orthogonal and intersecting cut planes in **(a,d,g)**. The coloured ellipsoids give the preferred orientation direction of the HA crystallites *c*-axis based on directional HA002 scattering. The ellipsoid volumes are scaled with the intensity, while the elongated shape and colour both indicate the Hermans’ parameter *S*. *Prox.* proximal, *cc* cartilage canal.

**Figure 4:**
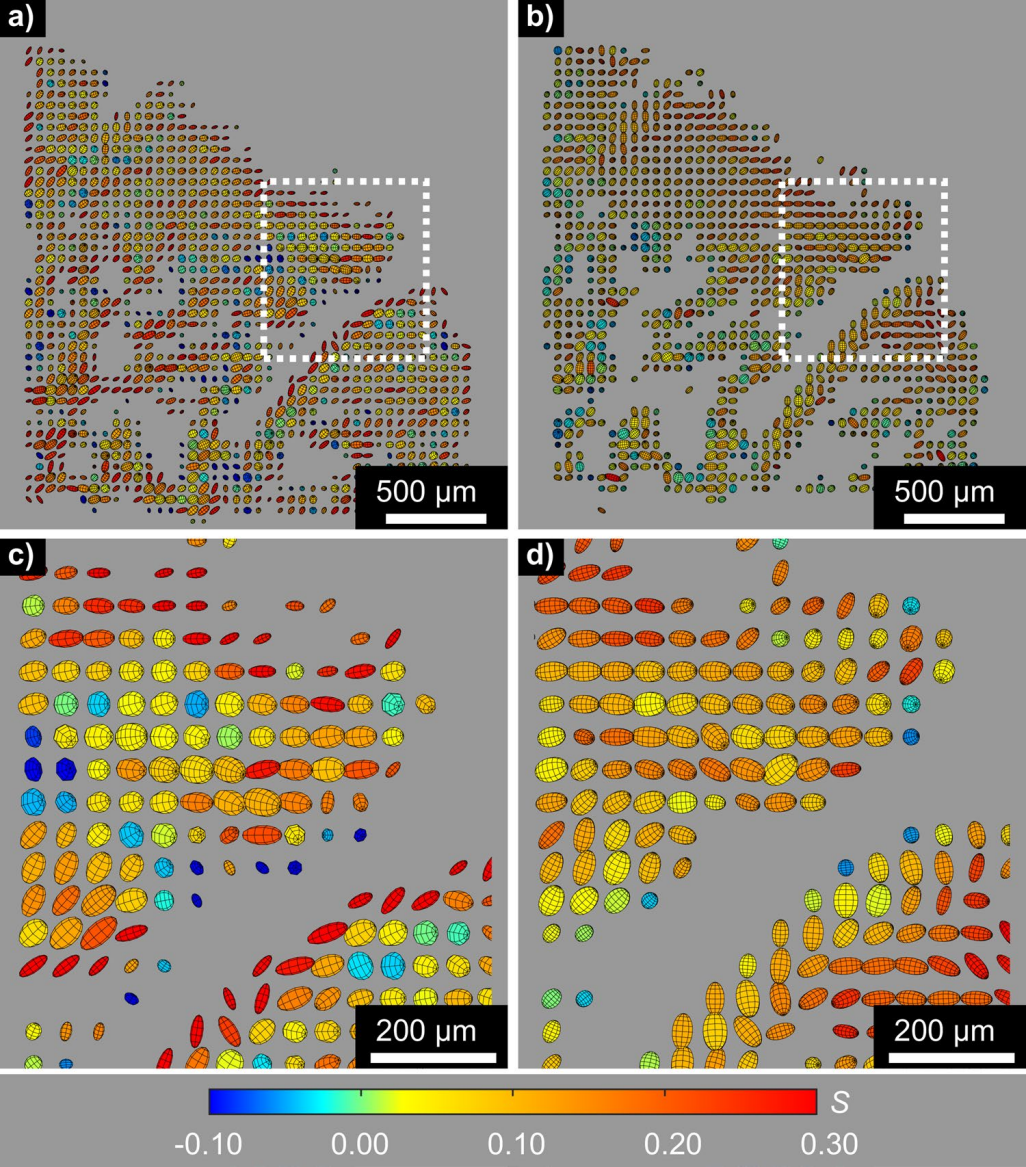
Comparison of **(a,c)** XRDTT based on HA002, and **(b,d)** SASTT. The depicted cross-sections show the same region as in Figs. 2a and 3a. **(c,d)** are magnified sections of **(a,b)**, respectively. The preferred HA crystallite orientation direction associated with each voxel is indicated by ellipsoids. The shape elongation and colours are scaled by the Hermans’ parameter *S* and the volume is scaled by the scattered intensity. Note the systematic differences between the reconstructions, most markedly towards the surfaces.

Performing tensorial tomography analysis of the weakly textured HA002 diffraction data, we could for each voxel within the bone and mineralized cartilage region obtain the local preferred orientation of the unit cell *c*-axis of the HA crystallites. In the XRDTT reconstruction procedure (cf. “Materials and methods”) a uniaxial texture model based on spherical harmonics was assigned to each sample voxel^40,41,56^. The resulting orientational maps were 3D registered with the corresponding PPC-CT tomograms to relate the HA orientation maps to the bone and cartilage morphology. Co-registered PPC-CT and XRDTT data are shown in Fig. 3 for three intersecting and orthogonal cross-sections through the sample. Magnified views are provided to visualize the local preferred orientation. The preferred orientation direction of the HA *c*-axis in each voxel is indicated by the direction of oriented ellipsoids, whose volumes scale with the reconstructed isotropic scattered intensity. We used Hermans’ orientation function *S*(**r**') ≡ ½(3 < cos^2^ Θ(**r'**) > -1), also known as a uniaxial order parameter, to provide a normalized measure of preferential oriented crystallites per voxel^57,58^. *Θ*(**r'**) can be considered the angle a given crystallite deviates from the preferred orientation axis in the voxel at **r**', and < … > denotes average. For regions with full alignment of the scattering with the predominant orientation axis, *S* tends to 1. For full isotropy, *S* is 0. If the scattering is directed perpendicular to the predominant orientation axis, *S* takes the value − 1/2. A more detailed description of the Hermans’ distribution function and the relation to the reconstructed reciprocal space maps is given in the SI (cf. S4). Quantitative information about the variation of *S* with position is provided in Figs. 3 and 4, represented by the colour coding and shape of the oriented ellipsoids. For *S* > 0, the ellipsoids are prolate, for *S* < 0, the ellipsoids are oblate, while for *S* = 0 the ellipsoids are spherical.

The values for *S*_HA002_ were found to be predominantly in the range of 0 to 0.3, indicating a low to moderate degree of preferentially oriented crystallites in all mineralized regions of the sample. Throughout the whole sample slow directional variations of the HA *c*-axis orientation were found. We discovered a tendency of the HA crystallites to point towards the bone-cartilage interface, as shown in Fig. 3a,d,e. In regions where cartilage canals enter into the bone (Fig. 3b,c,f,i) we observed distinct patterns of HA orientation. In Fig. 3b,f it is seen that near canals, the HA crystallite *c*-axis tends to deviate towards the cartilage canals, whereas the HA crystallites tend to orient parallel to the canal wall deeper into the bone, shown in Fig. 3c. Evidently, the HA crystallites gradually change orientation direction with increasing distance from the incorporated cartilage canal over a distance of approximately 200–300 µm, as shown in Fig. 3c,f.

While the experiment was primarily designed for wide-angle XRDTT, texture was also observed in the SAXS signal from the mineralized collagen fibrils^30^ in the bone and mineralized cartilage regions, cf. Figure 4. We note that there was also small-angle scattering originating from the unmineralized growth cartilage regions of the sample, as shown in Fig. 2h, but our experiment was not optimized for capturing and analysing this SAXS signal. It is reasonable to assume that the strongly oriented small-angle scattering in the bone region (cf. SI) originates from the HA platelets since the small-angle scattering was found to originate from the same regions as the HA002 wide-angle diffraction, see also Refs.^11,30,40,41^. The small-angle scattering was predominantly perpendicular to the directional HA002 diffraction (cf. SI, Figure S4.1), supporting the assumption that the small-angle scattering originates from the refractive index difference between the HA crystallites and the surrounding organic matrix^30^. These considerations imply that the reconstructed preferred orientation maps of the HA crystallites based on shape (SAXS) or HA002 (XRD) would be expected to be the same. Indeed, SAXS tensor tomographic reconstruction of the small-angle signal (using SASTT, see Refs.^40,41^) provided complementary 3D orientational maps to those obtained by wide angle XRDTT, see Fig. 4 and Discussion.

## Discussion

Our motivation for this study was to elucidate spatial variations of the bone mineral composition and orientation close to the ossification front in growing bones. To this end, we obtained samples from the femoral epiphysis of a piglet with high breeding value for articular osteochondrosis. Specifically, a condylar and a trochlear sample from the same distal femoral epiphysis were investigated using the novel imaging method wide-angle X-ray diffraction tensor tomography (XRDTT). In this work we present primarily the results from the condylar sample, in the form of 3D carbonated hydroxyapatite (HA) orientation maps. Similar results for the trochlear sample (cf. SI) will not be discussed further here.

XRDTT relies on numerical 3D reconstruction of anisotropic scattering from wide-angle diffraction patterns, as recently published also by Grünewald et al.^16^. An important point in our approach was to exploit the possibility offered by X-ray imaging of measuring a hydrated sample, to ensure that both the bone and cartilage regions remained close to their natural state. Another issue we consider crucial is that for reaching a better understanding of bone and cartilage, microscopic details must be obtained over a sufficiently large sample volume to study structural variations around functional biological features, such as the mineralized cartilage, the primary and secondary spongiosa and cartilage canals close to the ossification front. Hierarchical imaging with XRDTT should be ideal for this purpose, allowing either mineral or orientation distributions of nano-crystallites to be studied at a voxel size below 1 µm^16^, or to provide a panoramic overview over comparably large (~ mm^3^) sample volumes^40,41^.

By the (isotropic) XRD-CT reconstruction procedure, we obtained spatially resolved diffractograms over a large (0.2–6.0 Å^−1^) *q-*range for the whole sample. Based on the reported structure of HA^55^ all the diffraction peaks from the mineralized sample regions could be indexed as originating from HA, cf. SI. The HA Bragg peaks were broad and overlapping, consistent with previous reports^4,48^. One common explanation for the broad peaks is that HA is known to appear in a range of different, yet closely related structural modifications, typically by ionic substitution, thereby giving slight variations of the unit cell parameters^48^. From the integrated HA002 peak intensity, we retrieved 3D density maps of HA, shown in Fig. 2f and in the SI, closely resembling the high-density regions observed with PPC-CT. While we did not detect any other minerals with XRD-CT, we note that this could be due to weak signals or the fact that the sample studied had been formalin-fixed and stored on ethanol for approximately 12 weeks before the experiment, possibly destroying metastable precursor phases^59^.

The radial width (Δ*q*) of the HA002 diffraction peak decreases towards the ossification front (Fig. 2g). *If* assuming that the variations in peak width are caused by variations in the crystallite length, Williamson-Hall analysis^51^ provides estimates of the average HA crystallite length in the cross-section in Fig. 2g of 356 ± 84 Å, using a crystallite shape factor of *K* = 0.9 (Ref.^60^), and that crystallites with lengths up to ≈550 Å are found close to the ossification front. The analysis revealed a uniform strain of about 0.003 ± 0.001, cf. SI. A recent study on murine fetal bone reported *longer*^61^ crystallites found in the newly formed bone from endochondral ossification, than found in the mature bone, while other groups have previously reported *shorter* crystallites found close to the ossification front related to bone growth from either endochondral ossification in human fetal bone^48^ or intramembranous ossification^60^. Combined with the uncertainties in the XRD-CT analysis, we prefer to avoid (over-) interpreting the observed changes in HA peak width as crystallite length variations.

Weak scattering was observed from the cartilage regions in the sample, and some Bragg peaks were observed in the cartilage in the range *q* = 0.2–0.6 Å^−1^ (cf. Fig. 2h). These peaks were not observed in the mineralized cartilage and bone regions, likely due to the strong small-angle scattering from HA extending up to *q* ≈ 1.0 Å^−1^. The peak at *q* = 0.49 Å^−1^ is known to originate from the equatorial scattering of collagen fibrils^62,63^. Within the growth cartilage zones, subtle differences in the Bragg peak intensities in the *q*-range 0.4–0.6 Å^−1^ were observed between the resting and hypertrophic zones (cf. Fig. 2h). As the chondrocytes volume increases in the hypertrophic zone, there is a difference in cell/collagen matrix ratio^2^, possibly explaining the difference of the Bragg peak intensities. Near the bone-cartilage interface, the HA mineralization surrounding the cartilage canals continued approximately 200 µm into the cartilage, in agreement with previous findings^64^. Studies by second harmonic generation and two-photon fluorescence microscopy on physically cut and decalcified samples report fluorescence from regions surrounding cartilage canals close to the mineralized zone in the ossification front, and it has been suggested that the fluorescence could be associated with mineralization^65^. Our XRD-CT results give direct evidence that the mineralized structure extending into the growth cartilage surrounding the cartilage canals is HA (Fig. 2h).

XRDTT enabled the study of local variations of HA crystallite orientation in the subchondral bone and mineralized cartilage. 3D image registration of the XRDTT orientation maps with the PPC-CT tomograms (Fig. 3) showed a close correspondence between these two complementary techniques, allowing us to relate the reconstructed HA orientation to the sample morphology. For the XRDTT reconstruction using the HA002 peak (cf. Figs. 3, 4a), the Hermans’ orientation function *S*(**r'**) was found to vary in the range 0–0.3 consistent with weakly oriented crystallites. The SASTT reconstruction (cf. Fig. 4b) provided *S*(**r'**) in the range − 0.15 to 0, indicating an equatorial scattering distribution. The Hermans’ parameters of equatorial reflections are related to the corresponding meridional reflections by a factor of − 2, which has thus been multiplied into the distributions in Fig. 4b,d to facilitate comparisons. Gratifyingly, the gross features of the XRDTT and SASTT reconstructions resemble each other, with the overall uniaxial orientation tending towards the bone-cartilage interface. Still, there are important and interesting differences, most pronounced towards the surfaces. Around cartilage canals entering the subchondral bone near the bone-cartilage interface we observed the HA crystallites tending to orient with their *c*-axis pointing towards the canals (Fig. 3b,f). Conversely, the *c*-axis is seen to orient parallel to the canal walls in regions deeper into the bone (Fig. 3c). This could be an adaption of the bone matrix due to the incorporation of cartilage canals into the ossification front^18,64^. In the recent article by Grünewald et al.^16^, they describe deviations between the XRD and SAXS based tomographic reconstructions, and speculate that the old truth of the crystallographic *c*-axis coinciding strictly with the long axis of the crystallites might need revision. Despite the coarse resolution and the low signal-to-noise ratio of our experiment, a possible explanation for the deviations we observe (cf. Fig. 4) is that similar mechanisms play a role. Deep into the bone, we observe that both the HA crystallite *c*-axis (from XRDTT) and the crystallite symmetry axis (from SASTT) appear to the directed towards the bone-cartilage interface, cf. Fig 4. Whereas a detailed assessment of the relation between the HA crystallite orientation and the spongiosa morphology was outside the scope of this study, a simple orientation analysis of the bone morphology based on the PPC-CT data is provided in the SI, S8. Indeed, also the PPC-CT analysis supports that the spongiosa has a directional component towards the bone-cartilage interface.

While we were able to explain and model most of the orientational features of the HA scattering, there are stripe-like features in the measured projections (cf. SI, Figure S4.3) that could not be observed in the reconstructed orientational tomograms. The tensorial tomography approach used in this work assigns a unidirectional distribution of preferred orientation per voxel, which might be problematic as the true sample could include multiple orientations within the relatively large (50 μm)^3^ voxel volume^16^. The voxel size of the reconstructed XRDTT data was 50 µm, defined by the width of the collimated X-ray beam, and chosen as a compromise between high resolution and a wide field of view. While we could observe distinct patterns of orientation in different bone regions, the characteristic dimensions of the trabeculae and matrix lacunae in the primary spongiosa and mineralized zone are comparable to the voxel size, precluding the capturing of finer details. Moreover, a uniaxial distribution is assumed in the reconstructions, whereas a biaxial orientation (i.e., preferred orientation not only of the HA *c*-axis, but also of the perpendicular axes *a* and *b*) may give a more precise description, as recently speculated also by Grünewald et al.^16^.

It is an interesting question to what extent the observed orientation distributions of HA associated with each voxel are affected by the X-ray beam dimensions at the sample. Clearly, a broad beam covering regions of the sample with large variations of HA orientation can effectively give isotropic scattering due to averaging. However, previous studies reveal that HA displays wide orientation distributions already when studied at the smallest relevant volumes containing, say, 4–8 fibrils. This observation has been reported for wide-angle XRDTT mapping of lamellar bone with a sub-micrometre beam^16^ and for enamel in high-resolution TEM^66^, consistent with the plausible notion that the range of orientations spans the full hierarchy of bone structures. While the HA nanoplatelets within a fibril are constrained to be essentially parallel to the collagen axis, they most likely have rotation symmetry. In contrast, the mineral localized between the fibrils is presumably free to adapt random orientations.

Tensorial X-ray CT is an upcoming technique which at least with currently existing X-ray instrumentation can exclusively be carried out at synchrotron facilities. The measurement series reported here lasted approximately 26 h, disabling the study of a series of samples during the synchrotron beamtime. With the increase in X-ray beam brilliance by a factor 100 provided by the ESRF Extremely Brilliant Source (ESRF-EBS; the new low-emittance high-energy storage ring built in Grenoble), we expect the measurement time to drop by at least one order of magnitude. Rather than opting for a smaller sample than the ~ (2 mm)^3^ sample which gave the panoramic overview across several distinct bone and cartilage zones in the current study, the resolution can then be significantly improved, while still measuring the full sample during a practical amount of time.

In summary, we have studied HA orientation in growing bone in a femoral condyle of a young piglet using tensor tomography based on both XRD and SAXS. Tensor tomography provides non-destructive 3D orientational imaging, yielding information that to our knowledge would be difficult to obtain by tedious and destructive methods involving physical cutting or grinding of the sample^24^. The mineralized regions in bone and cartilage were found to consist solely of HA, with no indications of crystalline precursor phases. Wide field-of-view 3D maps showing the texture properties of the HA phase across the bone-cartilage sample complement the high-resolution images obtained by PPC-CT. The HA crystallites were found to be preferredly oriented, with large domains of similar orientation directed towards the bone-cartilage interface. The HA crystallite orientation in some regions is clearly influenced by the presence of cartilage canals. Our work contributes to the understanding of developing bone as an early case study of the bone ultrastructure near the ossification front.

## Materials and methods

### Sample

The current examined post-mortem material originated from a study carried out at the Norwegian University of Life Sciences, with full pre-approval of all experimental protocols from the Norwegian National Animal Research Authority (approval number: FOTS ID 2010/2630). Two sections of approximate dimensions 2.1 × 2.1 × 2.7 mm^3^ containing the bone-cartilage interface, i.e. the ossification front of the distal femoral epiphysis (bone end) of a 60-day old Landrace piglet were studied, one from the medial condyle and the other from the lateral trochlear ridge. Similar observations were made for both samples, and we have thus chosen to present the results from the condylar sample, see SI for some results from the trochlea. The presented sample included regions of both bone and growth cartilage. The articular layer of cartilage was physically trimmed to fit the sample within the field of view. The hind limb (cf. SI) was formalin fixed and kept in 70 wt.% ethanol, 30 wt.% water. The samples were mounted using cyanoacrylate in a custom sample holder, serving the purpose of keeping the sample hydrated during the measurements, while at the same time allowing near full transmission of X-rays.

### Propagation phase-contrast computed tomography (PPC-CT)

PPC-CT was performed at beamline ID15A^67^ at the ESRF—The European Synchrotron in Grenoble, France. A monochromatic beam with energy 50.00 keV was used, corresponding to a wavelength of λ = 0.2480 Å. Full-field illumination was used, with a rectangular beam cross-section of 7.6 × 3.5 mm^2^ flooding the sample. An area detector with 2401 × 1101 pixels (horizontal × vertical) and a square pixel size of 3.18 µm was used. The sample-detector distance was 2300 mm. The sample was rotated around a single horizontal tomography axis perpendicular to the incoming beam in 2001 steps with an angular range of *α* ϵ [0°, 180º]. An exposure time of 100 ms per projection was used. The total PPC-CT measurement time was approximately 4 min per sample. The recorded data was flatfield corrected before CT reconstruction using the built-in filtered backprojection in MATLAB. A Hann filter with frequency scaling 1.0 was used to reduce reconstruction noise. The reconstruction of the PPC-CT data took approximately 15 min on a desktop computer (Intel I7 CPU, 32 GB RAM, Nvidia GTX 1070 GPU).

### X-ray diffraction tensor tomography (XRDTT)

XRDTT was performed at ID15A, ESRF, Grenoble, France, during the same experimental campaign as for the PPC-CT measurements. A monochromatic beam with photon energy 50.00 keV (wavelength λ = 0.2480 Å) was used. The pencil-beam was collimated to 50 × 50 µm^2^ using compound refractive lenses and slits. The sample-detector distance was calibrated to 780 mm, and a Dectris Pilatus3 X CdTe 2 M 20-bit detector was used for collecting the 2D diffraction patterns. The detection area consisted of 1475 × 1679 pixels (horizontal × vertical), with a square pixel size of 172 µm. Momentum transfer values *q* ranging from 0.20 to 6.0 Å^−1^ were recorded. A CeO_2_ powder sample was used for calibration.

Measurements were performed by scanning the pencil-beam across the sample in (*x*,*y*) (Fig. 1b), while rotating the sample around the *y*-axis with angle *α* and the *x*-axis with angle *β.* and*.* A total of 65 × 67 points in (*x*,*y*) were collected per projection, with a step size of 50 µm in *x* and *y*. For each sample, 259 projections were measured for combinations of projection angles *β* ϵ [0°, 40º] and *α* ϵ [0°, 360º]. For *β* = 0°, equivalent to ordinary perpendicular-axis tomography, 61 projections with *α* ϵ [0°, 180º] were obtained. For *β* ϵ [5°, 40º] a reduced number of projections were measured compared to *β* = 0°. A total number of 65 × 67 × 259 = 1,127,945 diffraction patterns were collected. An exposure time of 50 ms for each diffraction pattern was used, giving a total exposure time of 15.2 h. With additional overhead time for motor movements, the total measurement time was approximately 26 h.

For the isotropic XRD-CT reconstruction (i.e. assuming no preferred orientation) the integrated diffraction patterns were azimuthally averaged. Diffraction patterns from all projection angles (*α*,*β*) were used in the CT reconstruction to maximize signal-to-noise ratio and to reduce artifacts from preferentially oriented crystallites^34,38^. Owing to the weak X-ray diffraction signal from cartilage, the diffractograms presented in Fig. 2h,i were averaged over regions of 3 × 3 × 3 voxels, i.e. a volume of 150 × 150 × 150 µm^3^, to get a sufficient signal-to-noise ratio. XRDTT reconstruction was done using the freely available small-angle scattering tensor tomography (SASTT) package^40,41^ developed by the CXS group, Paul Scherrer Institute, Switzerland. For more details regarding XRD-CT and XRDTT data reconstruction, cf. SI. The XRDTT/SASTT reconstructions were performed on a server with dual Intel Xeon Silver 4116 CPUs (24 cores) and 256 GB of RAM. Each full SASTT/XRDTT reconstruction lasted approximately 4 h. Scalar XRD-CT reconstructions were done at the same system, with a reconstruction time of approximately 30 min.

### Ethics approval

No animal was euthanized specifically for this study. The animal originated from a previous study in which animal use was approved by the Norwegian National Animal Research Authority (belonging to the Norwegian Food Safety Authority) with reference number 2630. All animals were kept in accordance with the national guidelines and legislation (Animal Welfare Act 2009-06-19-97; Regulation for the keeping of pigs in Norway 2003-02-18-175).

## Supplementary Information

Supplementary Information.
