# Supplementary material for: Quantifying the hydroxyapatite orientation near the ossification front in a piglet femoral condyle using X-ray diffraction tensor tomography
Source: Sci Rep. 2021 Jan 25;11:2144. doi: 10.1038/s41598-020-80615-4 (PMC7835348; doi:10.1038/s41598-020-80615-4)
Supplement: Supplementary file 1 — Supplementary Information. [file 41598_2020_80615_MOESM1_ESM.pdf]

Supplementary Information

Quantifying the hydroxyapatite orientation near the ossification front in a piglet femoral condyle using X-ray diffraction tensor tomography

Fredrik K. Mürer[1], Basab Chattopadhyay[1], Aldritt Scaria Madathiparambil[1], Kim Robert Tekseth[1], Marco Di Michiel[2], Marianne Liebi[3], Magnus B. Lilledahl[4], Kristin Olstad[5], Dag W. Breiby[1,6*]

[1]PoreLab, Department of Physics, Norwegian University of Science and Technology (NTNU), Høgskoleringen 5, 7491 Trondheim, Norway
[2]ESRF - The European Synchrotron, 71 Avenue des Martyrs, 38000 Grenoble, France
[3]Chalmers University of Technology, Gothenburg, SE-412 96, Sweden
[4]Department of Physics, Norwegian University of Science and Technology (NTNU), Høgskoleringen 5, 7491 Trondheim, Norway.
[5]Faculty of Veterinary Medicine, Department of Companion Animal Clinical Sciences, Norwegian University of Life Sciences (NMBU), Equine Section, P. O. Box 369 Sentrum, 0102 Oslo, Norway
[6]Department of Microsystems, University of South-Eastern Norway (USN), Campus Vestfold, 3184 Borre, Norway

*Corresponding author: Dag W. Breiby (dag.breiby@ntnu.no)

S1 Sample

Two samples of approximate dimensions 2.1 × 2.1 × 2.7 mm^3 were cut from the right hind limb femur of a 60-day old/17 kg Landrace piglet for X-ray diffraction tensor tomography (XRDTT) and propagation phase-contrast computed tomography (PPC-CT) experiments. The animal originated from the Norwegian University of Life Sciences, Oslo, Norway. The current examined post-mortem material originated from a study carried out at the Norwegian University of Life Sciences, with full pre-approval of all experimental protocols from the Norwegian National Animal Research Authority (approval number: FOTS ID 2010/2630). The whole limb was fixed in 4% phosphate-buffered formaldehyde and later stored in a 70 wt.% ethanol 30 wt.% water solution. Both samples provided similar features, and therefore results from only one sample is presented in the article. The position of the presented sample relative to the femoral condyle is shown in Figure S1. A custom sample holder shown in Figure S1d was made by sealing a Ø3 mm polyimide ("Kapton") tube (Goodfellow Cambridge Ltd) with epoxy and cyanoacrylate ("super glue"). The sample was placed onto a rubber spacer to avoid scattering from the steel rod connecting the sample cell to the goniometer.

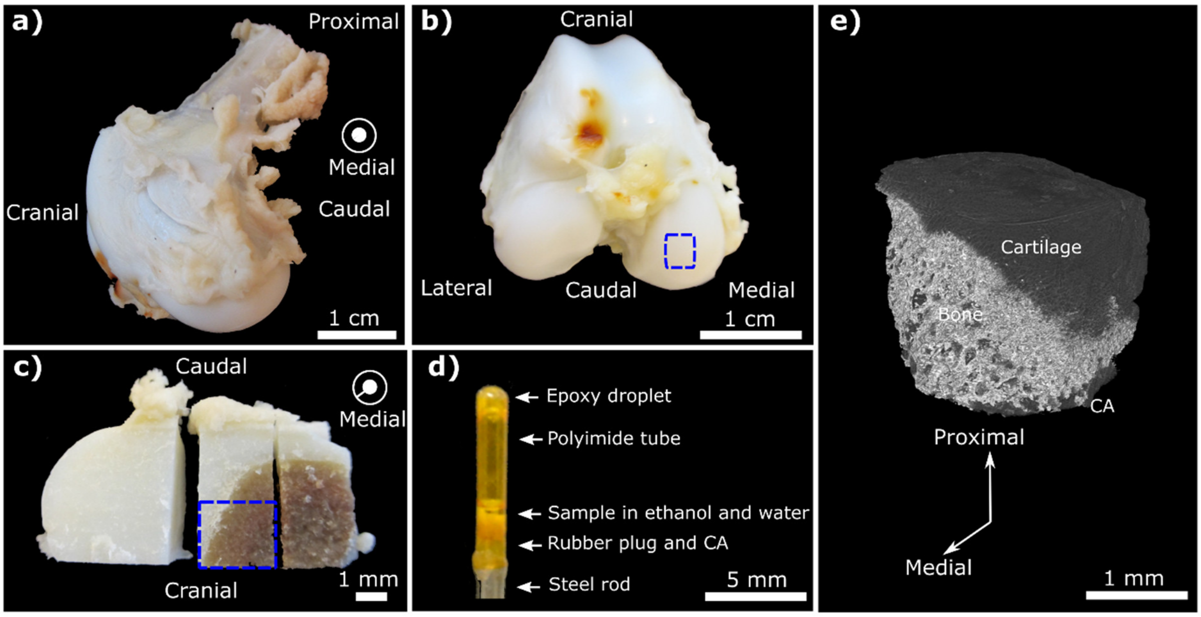

Figure S1 A porcine femoral condyle and the sample presented in the article. Blue dashed rectangles indicate the cut section of the sample presented. a) Distal end of a right hind limb femur from a 60-day old Landrace piglet. b) Orthogonal view with respect to (a). c) Cut section from the medial femoral condyle. d) Closed sample holder used for XRDTT and PPC-CT measurements. The sample holder steel rod end (Ø3 mm) was mounted onto a goniometer head. e) Reconstructed PPC-CT 3D sample volume. Abbreviation: CA: cyanoacrylate.

S2 XRDTT data collection

Figure S2.1a shows the geometry of the XRDTT experiment performed at ESRF ID15A, see also Refs. [1,2]. The sample was mounted on a goniometer translation and rotation stage, allowing sample translation laterally in x and y and rotation around the translation axes by angles α and β. The well-defined horizontal and vertical dimensions of a collimated “pencil” synchrotron X-ray beam of 50 × 50 μm^2 effectively define the voxel size of the XRDTT experiment. The sample was raster-scanned laterally (coordinates x and y) in a grid-pattern, and for each (x,y,α,β) position, the corresponding diffraction pattern was collected on a 2D detector. As for conventional X-ray diffraction experiments, the collected diffraction patterns contain structural information about the crystal structure and orientation of the scattering materials. Specifically, the intensity distribution $I(\mathbf{q})$ is proportional to the absolute square of the Fourier transform of the electron density distribution[3]. Density variations are systematically probed along the scattering vector $\mathbf{q} \equiv \mathbf{k}_f - \mathbf{k}_i$, where $\mathbf{k}_f$ and $\mathbf{k}_i$ denote the scattered and the incoming wave vectors, respectively. For elastic scattering, $k = |\mathbf{k}_f| = |\mathbf{k}_i| = 2\pi/\lambda$. The magnitude of the scattering vector is given by $q = |\mathbf{q}| = 4\pi\sin(\theta)/\lambda$, and for isotropic samples, the scattered intensity $I(q)$ depends only on q, not $\mathbf{q}$. The Bragg angle θ is half the total scattering angle 2θ. In the case of anisotropic scattering, information also about the orientation of the diffracting crystallites is contained in the intensity variations as a function of the detector azimuthal angle ϕ.

The number of scanning steps in x and y were 65 and 67, respectively, used for each of the 259 unique projections (combinations of angles α and β), giving a total number of measured diffraction patterns of 65 × 67 × 259 = 1,127,945. The diffraction patterns were integrated into 32 azimuthal sectors and 2048 radial bins using pyFAI[4]. After the radial and azimuthal averaging, the number of azimuthal sectors was further reduced to enhance the signal to noise ratio, which was particularly important for diffraction patterns obtained close to the edges of the sample and at the bone-cartilage interface. By assuming an effectively flat Ewald sphere at the energy used (E = 50.00 keV, $2\theta_{HA002} \approx 4.1°$), the HA002 diffraction pattern can be assumed to be point symmetric with respect to the beam centre and 32 azimuthal sectors were reduced to 16 sectors (cf. Figure S2.1d-e).

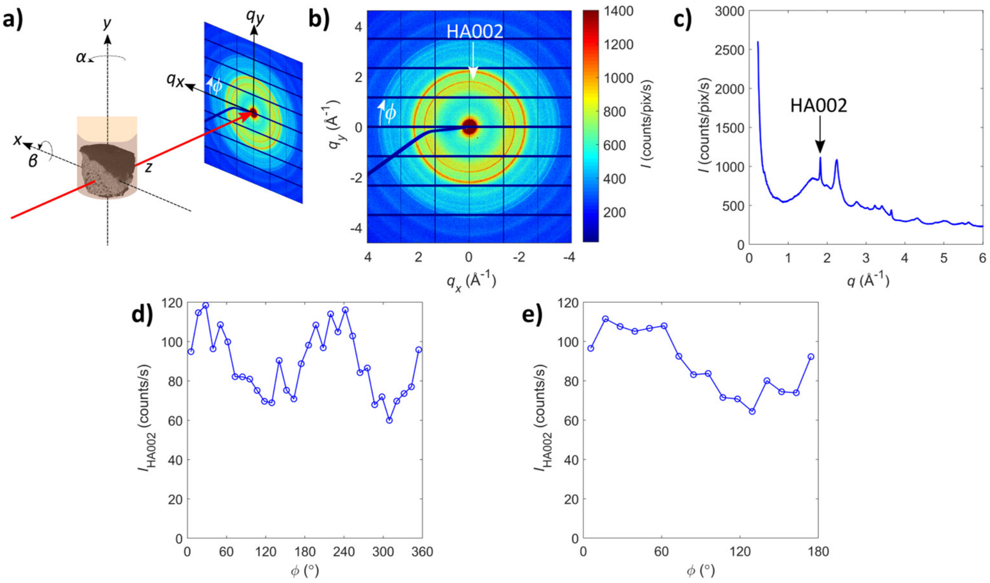

Figure S2.1 a) Illustration of XRDTT measurement setup. b) A recorded diffraction pattern originating from the bone region of the sample. Horizontal and vertical lines indicate detector gaps. The beamstop is seen as a shadow extending towards the lower right region. The intensity has been saturated at low q to increase the visibility of the HA Bragg peaks. c) Radially integrated version of the diffraction pattern presented in b). d) Azimuthal intensity variation of the fitted HA002 Bragg peak after background subtraction. The data was integrated in 32 sectors covering $\phi \in$ [0°, 360°). e) Reduction of azimuthal bins from 32 to 16 by assuming the diffraction pattern to be point symmetric with respect to the beam centre.

By spatially averaging all the ~1.1 million radially integrated diffraction patterns (cf. Figure S2.1c), one gets a "master" diffractogram representative of the whole sample including the sample holder and the ethanol and water solution, as shown in Figure S2.2. All the prominent Bragg peaks could be indexed to originate from HA and collagen in the bone/cartilage sample, and from cyanoacrylate (CA) and polyimide in the sample holder. The ethanol and water solution filling the tube provided a broad smoothly varying background signal. Note that there were additional Bragg peaks appearing in the cartilage regions of the sample (cf. Figures 2h and 2i in the main article), but these peaks were too weak to be visible when the diffractograms from the whole measured sample volume were averaged.

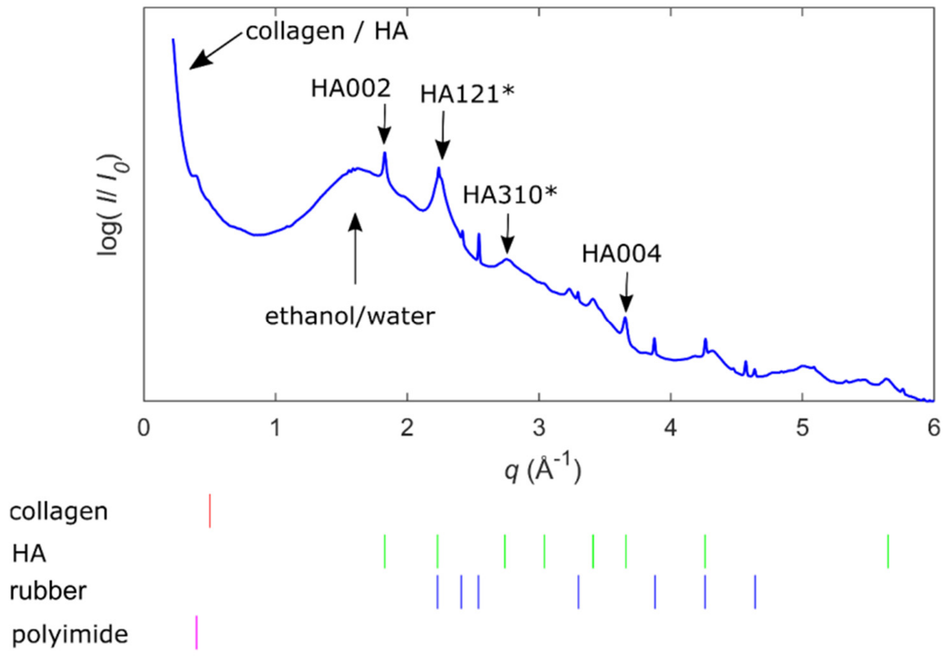

Figure S2.2 Spatially averaged radially integrated diffraction patterns from the whole sample including sample holder. Prominent Bragg peaks have been labelled. Abbreviations: HA – hydroxyapatite. HA121* consists of overlapping peaks HA121/211/112/202/300. HA310* consists of overlapping peaks HA310/130/212/122.

S3 Isotropic XRD-CT reconstruction

Isotropic XRD-CT reconstruction was done to obtain spatially resolved 3D chemical maps for the full sample volume. The full set of diffractograms was combined to generate separate 3D sinograms for each $q = |\mathbf{q}|$, which were then reconstructed to yield 3D tomograms. Tomographic reconstructions were done by using custom MATLAB macros based on the cSAXS MATLAB macros[5] and the ASTRA Toolbox[6,7], utilizing the 3D filtered backprojection (FBP) algorithm[8]. To improve the signal-to-noise ratio for the textured bone and cartilage and to mitigate reconstruction artefacts caused by the anisotropy of the sample, we used projections obtained for all sample orientations (α,β) in the FBP reconstruction. Before isotropic XRD-CT reconstruction, an iterative alignment algorithm was used to horizontally and vertically align projections with subpixel resolution. Diffraction patterns from all obtained 259 projections were used in the FBP reconstruction to minimize noise, and a Ram-Lak filter with frequency scaling 1.0 was used. The sample attenuation was low (cf. S5) and therefore no attenuation correction was applied. The reconstructed voxel size in XRD-CT was 50 µm, defined by the scanning step size in (x,y), cf. Figure S2.1a. For validating the XRD-CT reconstruction procedure, a comparison of the *measured* diffractograms and the *reconstructed* diffractograms from different sample regions was made, cf. Figure S3. Figure S3b shows azimuthally averaged measured diffraction patterns obtained from different regions of the sample holder. The reconstructed diffractograms from the different regions (cf. Figure S3d), shows that while the main features of the HA and cartilage scattering appear to correctly be reproduced, artefacts originate from the sharp crystalline peaks of the rubber plug present at the bottom at the sample holder (cf. Figure S1d). Additionally, peaks from HA are detected in the reconstructed cartilage regions, whereas the measured scattering (cf. Figure S3b) shows that the cartilage regions far away from the bone regions do not contain any trace of HA.

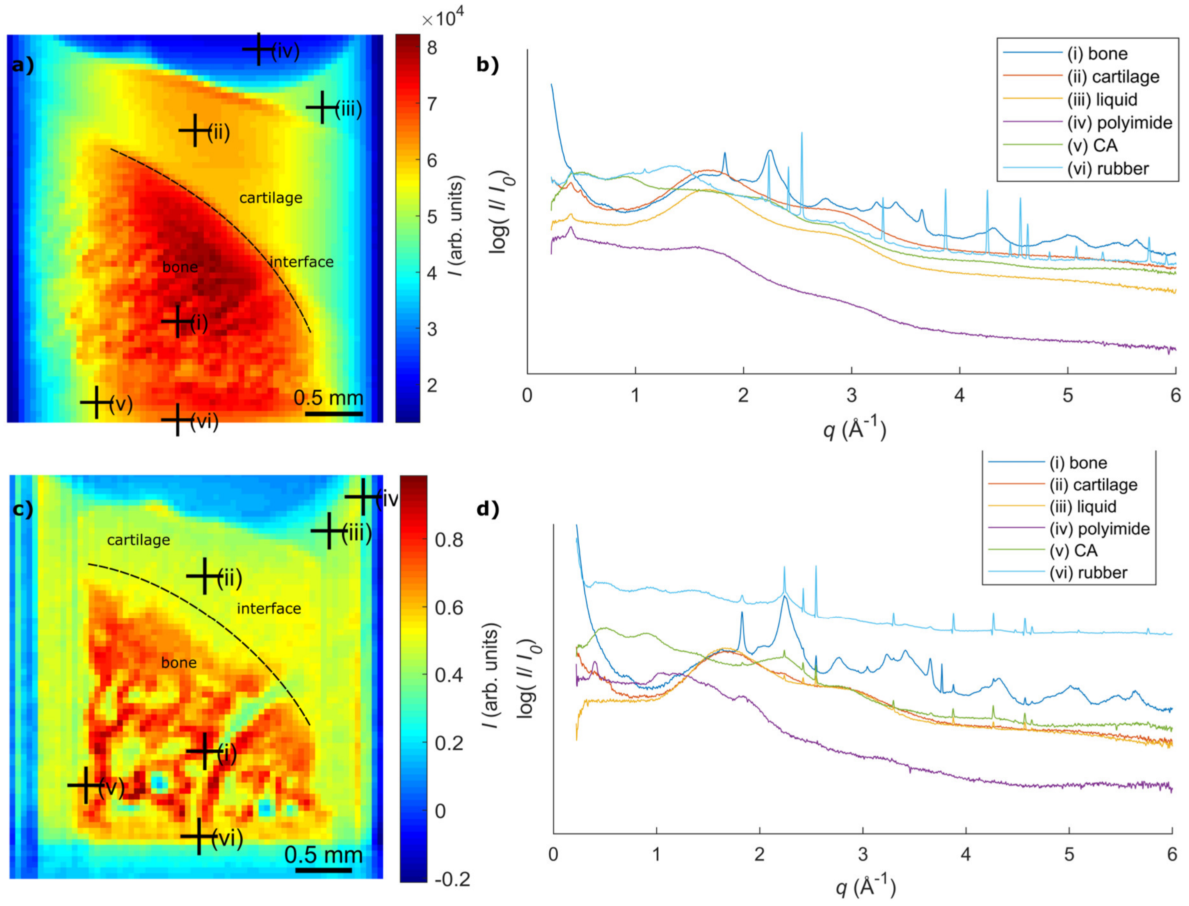

Figure S3 Comparison of a measured projection and a reconstructed cross-section from isotropic XRD-CT. a) Measured projection $I_n(x,y,q,\phi)$ for sample orientation (β = 0º, α = 40º), averaged over all q and all ϕ. b) Measured diffraction patterns at positions marked with a '+' in a), averaged over all ϕ. c) Reconstructed 2D cross-section from 3D XRD-CT reconstruction, going through the sample centre. The cross-section intensity is obtained by averaging over all q. d) Reconstructed diffractograms using FBP obtained from the positions marked with a '+' in c). Each curve originates from a volume of one voxel of size 50 × 50 × 50 µm^3.

S4 XRDTT and SASTT reconstruction

X-ray diffraction tensor tomography (XRDTT) reconstruction was done based on the HA002 Bragg peak. In addition, small-angle scattering tensor tomography (SASTT) reconstruction was done using the small-angle scattering originating from the HA crystallites in bone and mineralized cartilage[2] (cf. Figure S2.2). The anisotropic HA002 and small-angle scattering were observed to be approximately orthogonal, cf. Figure S4.1.

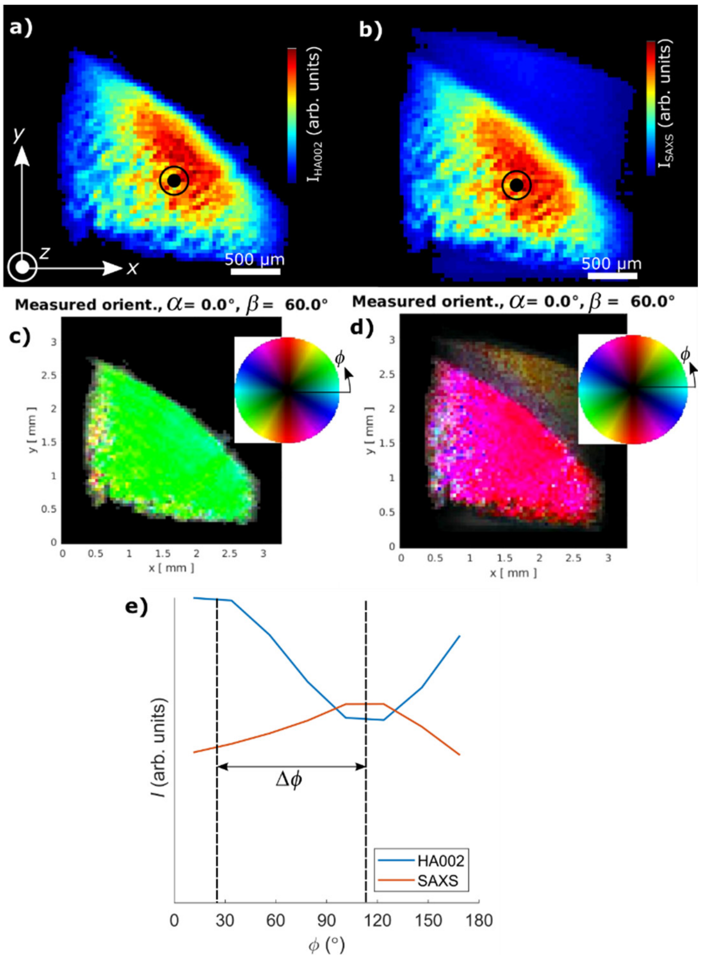

Figure S4.1 Orientational scattering from HA002 and the small-angle scattering (SAXS), studied for a single projection a) Fitted HA002 intensity after background subtraction, shown for a single projection with α = 0º, β = 60.0º. b) Fitted SAXS intensity for $q \in (0.2, 0.8)$ Å^{-1}. c) Dominating scattering direction for the fitted HA002 scattering, shown for the projection in a), obtained by Fourier analysis. d) Dominating scattering direction for the fitted SAXS signal, obtained for the projection in b). e) Azimuthal variation in scattering direction in the point marked in a) and b), obtained after background subtraction and fitting, and after assuming detector symmetry, to map the measured intensity to $\phi \in$ [0º, 180º), cf. Figure S2.1. Both HA002 and SAXS scattering have been plotted after binning into 8 azimuthal detector sectors. The dominating scattering direction of the small-angle scattering is directed $\Delta\phi \approx 90$º to the dominating scattering direction of the HA002 Bragg peak.

Pre-processing

For XRDTT the intensity contribution from the HA002 peak was selected by integrating the intensity for the HA002 peak in the range $q \in (1.791, 1.877)$ Å^{-1} and subtracting the background. The background contributions from the ethanol/water solution, air and the polyimide tube were estimated by visually determining the edges of the HA002 Bragg peak and subtracting the averaged intensity from each peak edge. This background subtraction strategy was compared with results obtained by fitting of the HA002 peak for each detector sector with a nonlinear fitting routine[9], cf. Figure S4.2. The simplified background subtraction approach used for intensity estimation of the HA002 peak provided a close match of the

results from the nonlinear fitting routine, and had the advantage of being substantially faster, and less prone to erroneous intensity values in the diffraction patterns obtained from the sample edges.

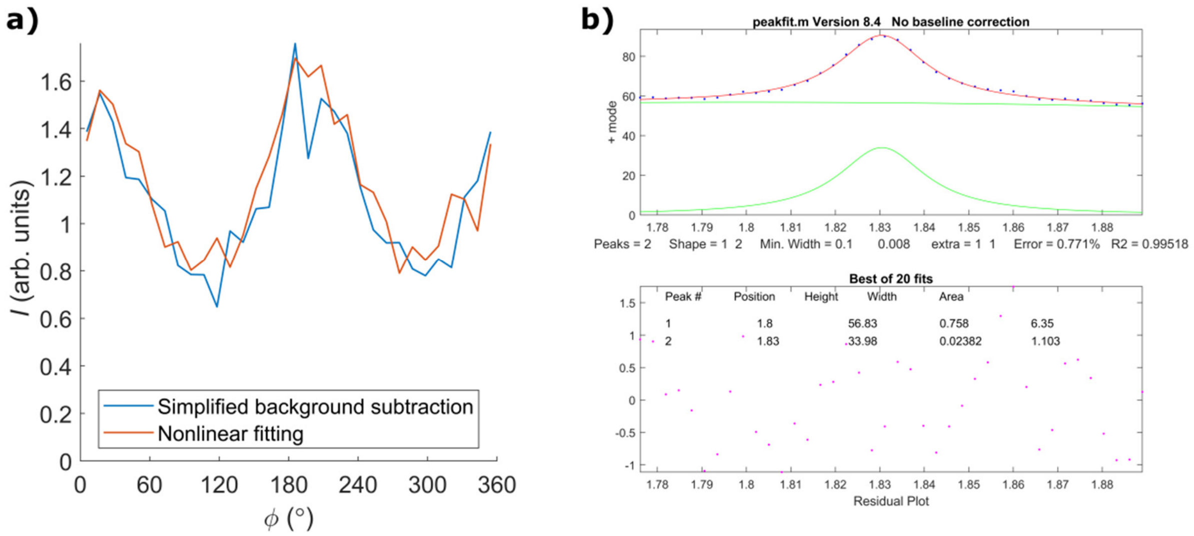

Figure S4.2 Background subtraction for XRDTT of HA002 illustrated for a single diffraction pattern obtained from the sample centre. a) $I_{HA002}(\phi)$ obtained by either (i) integrating the HA002 peak intensity and subtracting the background by visual inspection of the peak edges and (ii) a nonlinear fitting routine with modelling the HA002 peak as a Lorentzian peak with a Gaussian shaped background. b) Nonlinear fitting of intensity $I(q)$ of the HA002 peak for a single detector sector from a diffraction pattern. The background was fitted with a Gaussian peak (peak #1) and the HA002 peak (peak #2) was fitted with a Lorentzian peak.

For small-angle scattering analysis the intensity variations in the low q region close to the beam stop was fitted by a power law function $I(q) = Ae^{-Bq} + C$, with fitting coefficients A, B, and C, with B having values 3.98 ± 0.47 (arb. units). Due to detector gaps affecting several azimuthal detector sectors in the region where the small-angle scattering signal was studied, 8 azimuthal sectors covering $\phi \in [0^{\circ}, 180^{\circ})$ were used in the SASTT analysis, while 16 azimuthal sectors covering $\phi \in [0^{\circ}, 180^{\circ})$ was used for the XRDTT analysis of the HA002 peak.

Tensorial tomography reconstruction

XRDTT and SASTT reconstructions were done using the small-angle scattering tensorial tomography (SASTT) MATLAB package (software version 2020.01.24)[1,2] developed and provided by the CXS group at the Paul Scherrer Institute, Villigen, Switzerland. The description of the reconstruction procedure below closely follows Liebi et al.[2], with the exception of the Hermans' parameter S.

For each position $\boldsymbol{r'}$ in the sample coordinate system (each sample voxel), a *reciprocal space map* for a chosen q-range (i.e. by using small-angle scattering or Bragg peak HA002) was defined by

$$\widehat{R_q}(\boldsymbol{r'}) = \left| \sum_{l,m} a_l^m(\boldsymbol{r'}) Y_l^m[\Theta(\boldsymbol{r'}), \Phi(\boldsymbol{r'})] \right|^2, \qquad \text{(S4.1)}$$

where a_l^m are coefficients for spherical harmonics Y_l^m of degree l and order m. Note that in this description, the coefficients a_l^m have the dimension of square root of intensity. The input arguments of Y_l^m, $\Theta(\boldsymbol{r'})$ and $\Phi(\boldsymbol{r'})$, are polar and azimuthal angles, respectively. In both cases, i.e. XRDTT of HA crystallites using the HA002 peak and SASTT using the small-angle scattering from the platelets, uniaxial ("fibre") symmetry of the HA platelets was assumed, giving $m = 0$ in Equation S4.1. The angles $\Theta(\boldsymbol{r'})$ and $\Phi(\boldsymbol{r'})$ are related to the sample coordinates $\boldsymbol{r'}$ by

$$\begin{pmatrix} \sin\Theta(\boldsymbol{r}')\cos\Phi(\boldsymbol{r}') \\ \sin\Theta(\boldsymbol{r}')\sin\Phi\,(\boldsymbol{r}') \\ \cos\Phi\,(\boldsymbol{r}') \end{pmatrix}$$

$$= \begin{pmatrix} \cos\theta_{\mathrm{op}}(\boldsymbol{r}')\cos\phi_{\mathrm{op}}(\boldsymbol{r}') & \cos\theta_{\mathrm{op}}(\boldsymbol{r}')\sin\phi_{\mathrm{op}}(\boldsymbol{r}') & \sin\theta_{\mathrm{op}}(\boldsymbol{r}') \\ \sin\phi_{\mathrm{op}}\,(\boldsymbol{r}') & \cos\phi_{\mathrm{op}}(\boldsymbol{r}') & 0 \\ \sin\theta_{\mathrm{op}}(\boldsymbol{r}')\cos\phi_{\mathrm{op}}(\boldsymbol{r}') & \sin\theta_{\mathrm{op}}(\boldsymbol{r}')\sin\phi_{\mathrm{op}}(\boldsymbol{r}') & \cos\theta_{\mathrm{op}}(\boldsymbol{r}') \end{pmatrix} \begin{pmatrix} \sin\theta'\cos\phi' \\ \sin\theta'\sin\phi' \\ \cos\theta' \end{pmatrix} \qquad \text{(S4.3)}$$

where the angles $(\theta_{\mathrm{op}}(\boldsymbol{r}'), \phi_{\mathrm{op}}(\boldsymbol{r}'))$ define the *local preferred orientation direction* at the sample coordinate $\mathbf{r}'$. θ' and ϕ' are the polar and azimuthal angles in the sample coordinate system. Estimation of $\widehat{\boldsymbol{R}_q}(\boldsymbol{r}')$ was done through an iterative optimization routine that minimized the difference between the *simulated* scattering from the sample $\widehat{I_{n,q}}(x,y,\phi)$ and the *measured* scattering $I_{n,q}(x,y,\phi)$. Here n denotes a single projection in a direction given by (α,β) (cf. Figure S2.1a), and q denotes a specific q-value corresponding to either small-angle scattering or HA002 wide-angle scattering. The simulated scattering $\widehat{I_{n,q}}(x,y,\phi)$ from the sample was calculated by

$$\widehat{I_{n,q}}(x,y,\phi) = \sum_z \left| \sum_{l,m} a_l^m(\boldsymbol{r}') Y_l^m\left[\Theta(\boldsymbol{r}')|_{\theta=\pi/2}, \Phi(\boldsymbol{r}')|_{\theta=\pi/2}\right] \right|^2, \qquad \text{(S4.4)}$$

where the first summation is done along the beam path z, cf. Figure S2.1a. The subscript $\theta = \pi/2$ on the arguments of Y_l^m indicates that the spherical harmonics were evaluated at a plane in reciprocal space perpendicular to the beam direction. This is equivalent to the assumption of a flat Ewald sphere at the high energy used. The error function to be minimized is defined as

$$\epsilon_q = 2 \sum_{n,x,y,\phi} \omega_{n,q}(x,y,\phi) \left\{ \left[\widehat{I_{n,q}}(x,y,\phi)\right]^{1/2} \quad \left[\frac{I_{n,q}(x,y,\phi)}{T_n(x,y)}\right]^{1/2} \right\}^2, \qquad \text{(S4.5)}$$

where $\omega_{n,q}(x,y,\phi)$ is a window function to mask invalid pixels in the projections and $T_{n,q}(x,y)$ is the measured transmission. In the case of the bone and cartilage sample studied, the sample attenuation was low (cf. S5) and hence $T_n(x,y)$ was set to 1, i.e. no sample attenuation corrections were made. Minimization of ϵ_q by optimization of a_l^m and $(\theta_{\mathrm{op}}, \phi_{\mathrm{op}})$ was done by using the conjugate gradient method.

In the work of Liebi et al., the degree of orientation $\rho(\mathbf{r}')$ was calculated as

$$\rho(\boldsymbol{r}') = \frac{\sum_{l=1}^{\infty} |a_l^m(\boldsymbol{r}')|^2}{\sum_{l=0}^{\infty} |a_l^m(\boldsymbol{r}')|^2} \in (0,1), \qquad \text{(S4.6)}$$

indicating the local ratio of oriented crystallites. A classical method for describing uniaxial degree of preferred orientation, is by the Hermans' parameter $S(\boldsymbol{r}')$, defined as

$$S(\boldsymbol{r}') = \frac{1}{2}(3 < \cos^2\Theta(\boldsymbol{r}') > \quad 1). \qquad \text{(S4.7)}$$

It is readily seen that for crystallites having the axis highly aligned along the symmetry (preferred orientation) axis, S tends to unity. Similarly, S equals zero for isotropically oriented crystallites, and S = -0.5 in the case that the crystallite axes are perpendicular to the symmetry axis. Compared to the approach of S4.6, S has the virtue of better discriminating between meridional and equatorial distributions and was thus used in the present study. Because the high photon energy used in the experiment gives small scattering angles, no Lorentz or polarization corrections were made. Reconstruction parameters used in XRDTT and SASTT are provided in Table S4.1.

Table S4.1: Tensorial tomography reconstruction parameters. The steps and parameters are named after the entries in the SASTT MATLAB package.

STEP	Parameter	XRDTT using HA002	SASTT
Symmetric intensity	regularization	0	0
	regularization_angle	0	0
	itmax	20	30
SH angles	regularization	0	-
	regularization_angle	0	-
	itmax	50	-
	a1_init	1	-
	a2_init	12	-
	a3_init	10000	-
	l	[0 2 4]	-
	m	[0 0 0]	-
	theta_init	pi/2	-
	phi_init	pi/2	-
SH coefficients	regularization	1	1
	regularization_angle	0	0
	kernel3D	window3(3,3,3,@hamming)	window3(3,3,3,@hamming)
	itmax	20	30
	l	[0 2 4 6]	[0 2 4 6]
	m	[0 0 0 0]	[0 0 0 0]
	a	[0.001 0.0001 0.001 0.0001]	[0.001 -5 0.001 0.0001]
Combination of all parameters	itmax	50	500
	regularization	0	0
	regularization_angle	1	1
	regularization_angle_coeff	0	20

By comparing the measured projections with the forward-simulated projections of the reconstructed 3D model, a validation of the reconstruction could be made. Figure S4.2 shows comparisons of measured and simulated orientational scattering in tensorial tomography. A close match between the measured scattering and simulated scattering (cf. Equation S4.3) from the XRDTT model can be observed.

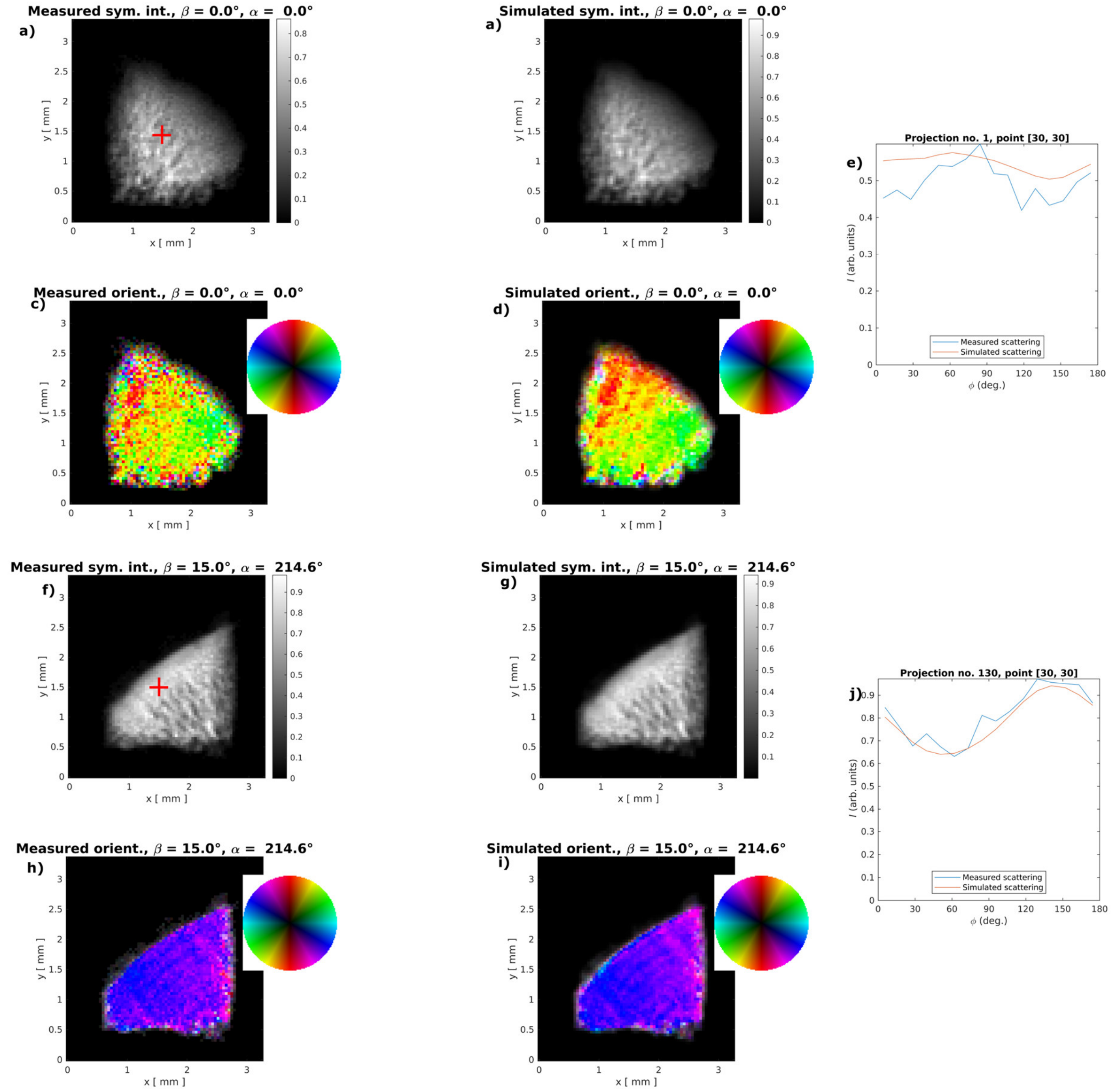

Figure S4.3 Comparison of measured and forward-simulated projections for the XRDTT model from the HA002 Bragg peak. a-b) Measured and simulated symmetric (isotropic) intensity. c-d) Measured and simulated orientation. The colour coding indicates the in-plane direction of preferred orientation, while the hue indicates degree of orientation (using Equation S4.6). e) Plot of azimuthal intensity variation $I(\phi)$ for point marked by a red cross in (a). The intensity variations are displayed for 8 sectors, $\phi \in [0^{\circ}, 180^{\circ})$ while 16 sectors, $\phi \in [0^{\circ}, 180^{\circ})$ were used for the final analysis of the HA002 scattering. f-j) Similar to a-e) for a different sample rotation angle (α, β).

To test the reproducibility of the reconstruction method, XRDTT of HA002 reconstructions were performed with different choices of initial guesses of the preferred orientation directions $\theta_{op}(\mathbf{r}')$, $\phi_{op.}(\mathbf{r}')$ and the spherical harmonic coefficients $a_l^m(\mathbf{r}')$. For SASTT, the experimental setup prevented capturing the full SAXS signal due to overlap with the detector module gaps, beamstop and beamstop support, cf. Fig. 2.1b. To ensure convergence of the SASTT reconstruction, the initial values for $\theta_{op}(\mathbf{r}')$ and $\phi_{op.}(\mathbf{r}')$ were assigned to the $\theta_{op}(\mathbf{r}')$ and $\phi_{op.}(\mathbf{r}')$ found from XRDTT reconstruction of HA002, as both the HA002 and SAXS signals were assumed to be caused by the directional scattering of HA crystallites. Thereafter the spherical harmonic coefficients $a_l^m(\mathbf{r}')$ for l = 2, 4, 6, m = 0 were optimized with penalizing positive values of $a_2^0(\mathbf{r}')$. Finally, all angles and coefficients were optimized with no constraints, giving the final reconstructed SASTT tomogram shown in Fig. 4 in the article.

XRDTT/SASTT allows the use of regularization on the angles $\theta(\mathbf{r'})$ and $\phi(\mathbf{r'})$ describing the local direction of preferred orientation. The angular regularization controlled by μ penalizes abrupt changes in local preferred orientation[2], and is implemented by adding an extra term to the error function (Eq. S4.5) and the gradients of the error function with respect to the optimization parameters, as described in detail in Liebi *et al.*[2]

An optimal choice of the regularization parameter μ is guided by (i) the plot of regularization error and data error as a function of the regularization parameter μ (Figure S4.4a). The region around the intersection of the two curves in Figure S4.4a indicates a suitable choice of regularization parameter, however the reconstructions would still need to be inspected visually. For the HA002 reconstruction, regularization provided artefacts indicating over-regularization (cf. Figure S4.4b), and therefore no regularization was done for the XRDTT tomograms presented in this study.

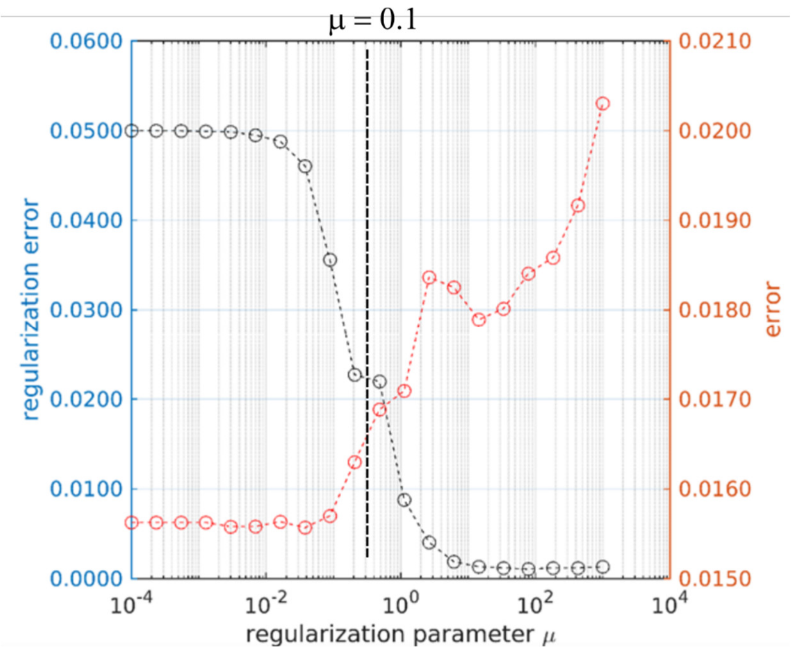

Figure S4.4 Angular regularization in XRDTT reconstruction of HA002. Plot of data error and regularization error as a function of varying the regularization parameter μ.

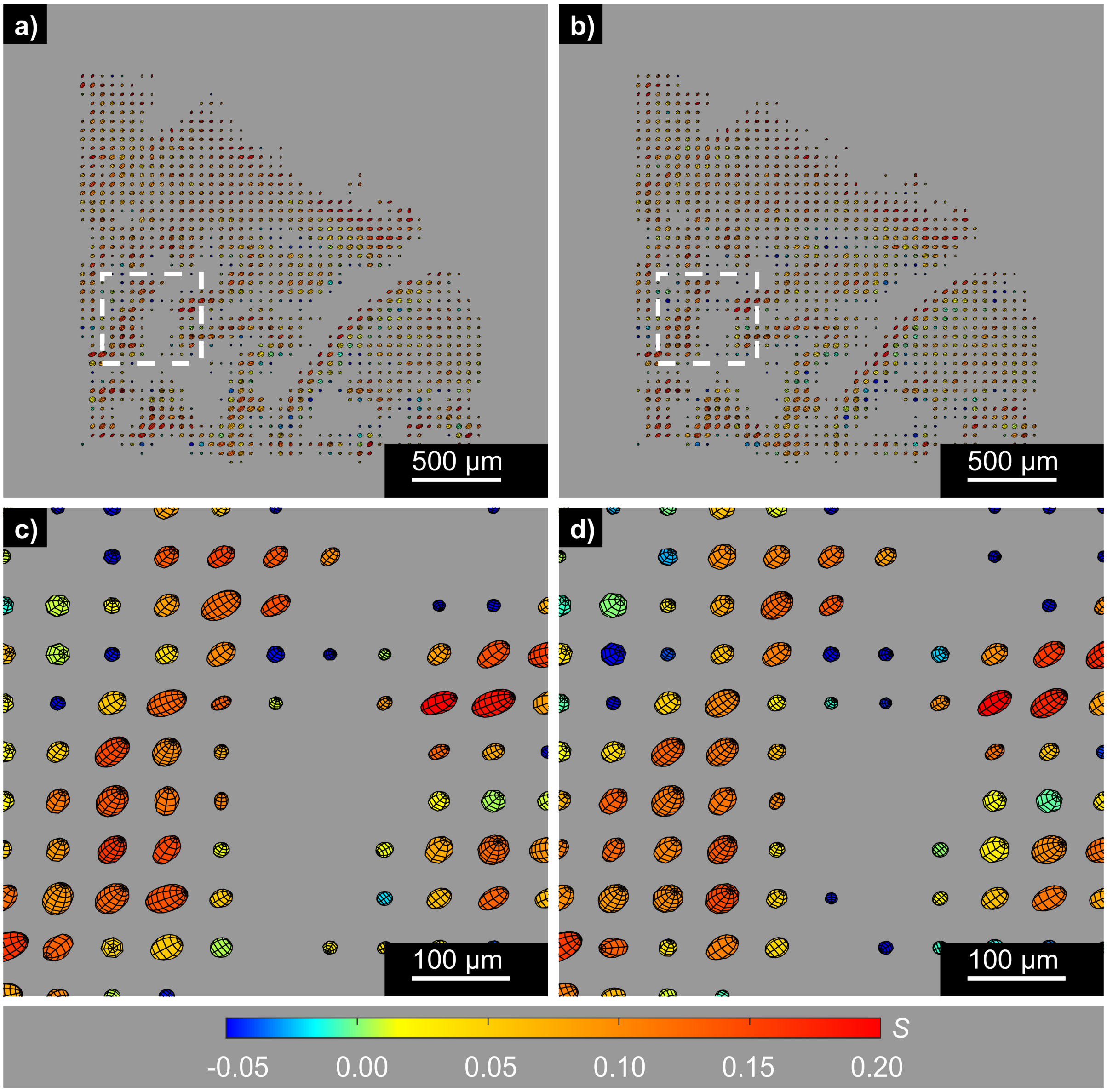

Figure S4.5 Effects of angular regularization in XRDTT reconstruction of HA002 displayed for the same cross-section as displayed in Figure 2a and 3a. a,c) No angular regularization, $\mu = 0$. b,d) Regularization with $\mu = 0.1$. Whereas $\mu = 0.1$ is close to the intersection of the two curves in Fig. S4.4, closer inspection of the magnified cross section suggests over-regularization. The ellipsoid volumes have been scaled by the reconstructed spherical harmonics coefficient a_0, while the shape and colours are scaled by the Hermans' parameter S.

Figure S4.6 shows how the orientational maps displayed in Figure 3, 4 and S4.5 relate to the reconstructed spherical harmonics coefficients a_0, a_2, a_4 and a_6 (cf. Equation S4.1), the reconstructed reciprocal space map for each voxel and the Hermans' parameter S, cf. Equation S4.7.

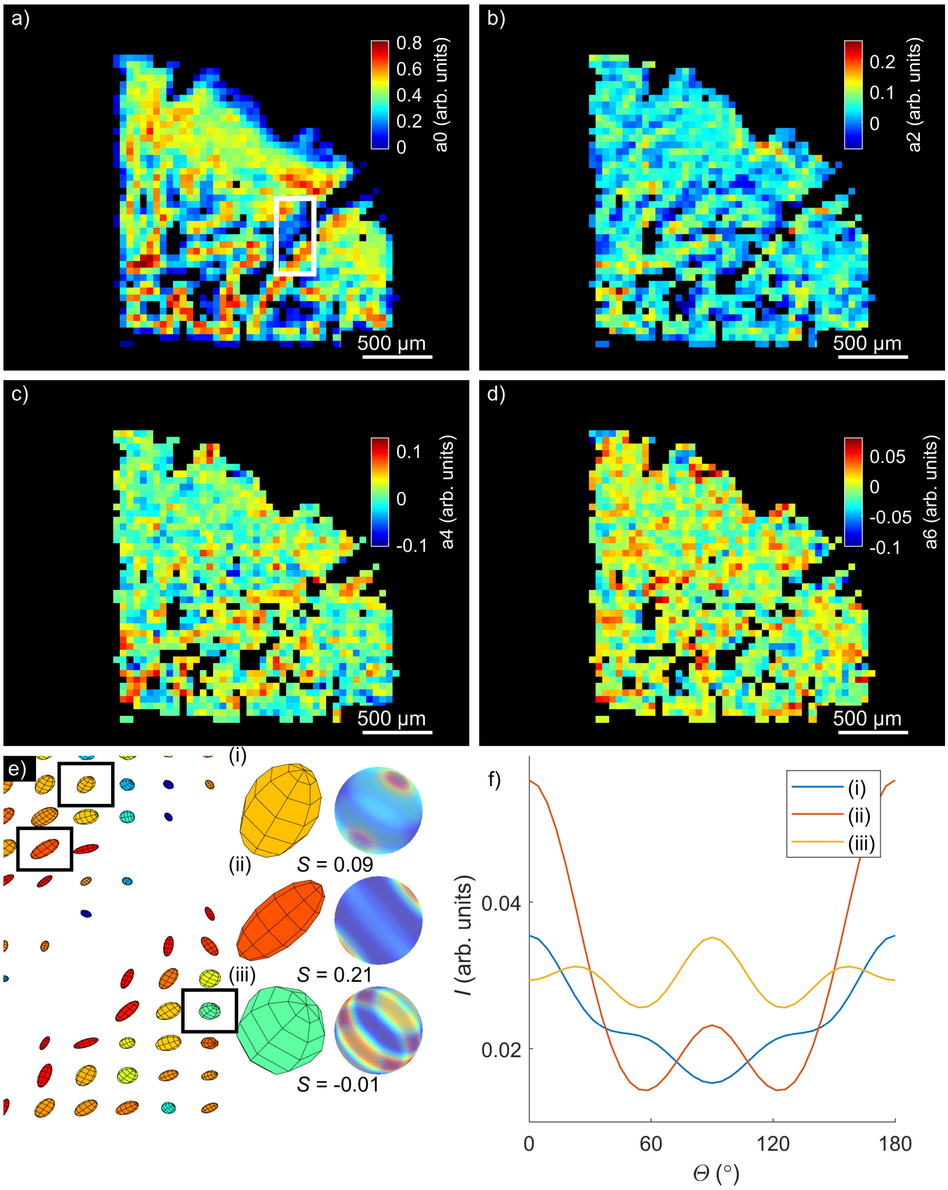

Figure S4.6 Reconstructed reciprocal space maps and spherical harmonics coefficients. a-d) Spherical harmonic coefficients for the cross-section of bone displayed in Figure 2, 3 and 4. The coefficients are a_l^m for l = 0, 2, 4 and 6 with m = 0 for all cross-sections. e) Local HA *c*-axis orientation displayed for a magnified section indicated with a rectangle in a). In addition to the ellipsoid representation used in Figure 3 and 4, the orientation and spherical harmonics coefficients are visualized as intensity distributions on spheres with radius q_{HA002} for three voxels (i)-(iii) indicated with boxes. The intensity distributions on the spheres have been calculated by using Equation S4.1. Note that the three spheres have been plotted with different colour scales, dependent of the maximum and minimum intensities. f) Polar intensity distribution for the selected voxels in e).

S5 Sample attenuation

Separate attenuation contrast CT-measurements was performed at ID15A to investigate the need for attenuation contrast corrections in the XRD-CT/XRDTT reconstructions. The attenuation-contrast experiment was performed immediately after the XRD-CT experiments and PPC-CT experiments, using a monochromatic beam with energy E = 50.00 keV, the same energy as in the XRD-CT experiments. Slits were open to provide a full-field beam with a rectangular cross-section of 7.64 × 3.50 mm^2. An area detector with 2401 × 1101 pixels and a pixel size of 3.18 µm was used. The detector was placed with a distance of 300 mm downstream from the sample, to detect sample attenuation without any phase-contrast effects. The sample was kept in the same sample holder as used for XRD-CT and PPC-CT (cf. Figure S1d) to ensure accurate assessments of sample attenuation. A single attenuation contrast projection of the sample is shown in Figure S5. For the detector pixels corresponding to the inside of the sample and sample holder (cf. Figure S5a), the average relative attenuation I_{att} / I_0 was calculated to be 4.6%, which was deemed to be sufficiently low to be neglected in the XRD-CT/XRDTT reconstructions. Still, attempts were made at setting the transmission T < 1 (cf. eq. S4.5), expectedly confirming that the reconstructions were not further improved.

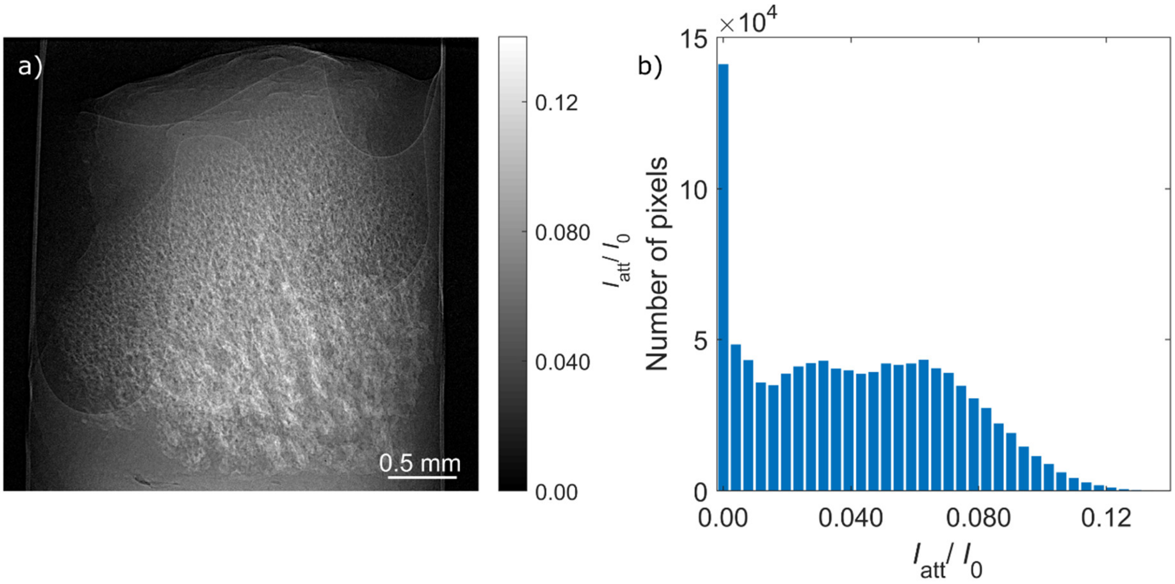

Figure S5: Sample attenuation. a) Synchrotron attenuation contrast CT projection obtained for sample orientation α = 0º, β = 0º. b) Histogram of the pixel values in a) showing the distribution of the relative attenuation values.

S6 Image registration

3D image registration to align XRD-CT and XRDTT tomograms with PPC-CT tomograms was done using custom MATLAB macros and the built-in function *imregister* which iteratively aligns the volumes in 3D using an optimization routine. As the 3D tensorial XRD-CT tomograms consisted of voxels of size 50 µm and the PPC-CT having voxels of size 3.18 µm, the XRD-CT tomograms were resized before registration. The datasets were manually pre-aligned to speed up convergence and to prevent the optimization routine from stopping in local minima. Before registration, a threshold and a morphological closing operation was done on the PPC-CT tomograms to remove cartilage regions of the sample and fine details of the trabecula slowing down convergence of the optimization. All datasets were binarized before registration. Registered cross-sections of the bone and cartilage sample are shown in Figure S6. A close correspondence of the PPC-CT and XRDTT tomograms is seen.

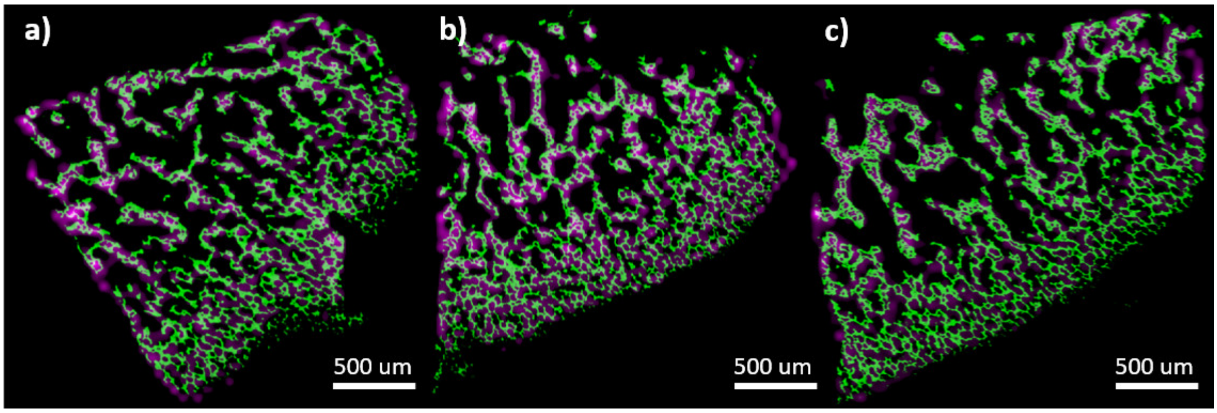

Figure S6: 3D image registration exemplified with orthogonal cross-sections chosen near the sample centre. Green: PPC-CT. Purple: HA002 XRDTT magnitude.

S7 Estimation of crystallite length and strain

The crystallite length and strain was estimated by using the Williamson-Hall approximation[10]. The peak broadening due to strain β_e can be approximated by

$$\beta_e = 2\epsilon\tan(\theta) \tag{S7.1}$$

where ϵ is the present strain and θ is the Bragg angle. The Scherrer equation[11] provides an estimate of the peak broadening due to crystallite size β_s . The Scherrer equation is given as

$$\beta_\mathrm{s} = \frac{K\lambda}{L\cos(\theta)} \tag{S7.2}$$

where λ is the X-ray wavelength and K is a constant depending on the crystallite shape. For estimates of the HA crystallite length, K was set to 0.9[12]. In the Williamson-Hall approximation one assumes that the total peak broadening β is a sum of the contribution from strain and crystallite size[10]

$$\beta = \beta_e + \beta_s. \tag{S7.3}$$

Using Equation 7.1 and 7.2 one gets

$$\beta\cos(\theta) = 2\epsilon + \frac{K\lambda}{L}, \tag{S7.4}$$

which is linear equation which can be used to estimate the strain ϵ crystallite length L if multiple Bragg peaks at position θ_I are measured with the corresponding peak widths in radians β_i. Before the calculations of Equation S7.4, the instrumental broadening was corrected. By fitting peaks in the CeO_2 powder diffractogram used for calibration, we estimated the instrumental broadening to $\Delta q = 0.0099$ Å^{-1}. The instrumental broadening estimated by the calibration sample was subtracted from the fitted sample data by[13]

$$\Delta q_{\mathrm{corrected}} = \sqrt{(\Delta q_{\mathrm{fitted}})^2 \quad (\Delta q_{\mathrm{calibration}})^2} \tag{S7.5}$$

The peak broadening due to the extension of the sample can for small scattering angles be estimated as[14]

$$\frac{\Delta q}{q} \approx \frac{d}{L} \tag{S7.6}$$

where Δq is the peak broadening of the peak centred at q, d is the sample thickness measured along the beam path and L is the sample centre-detector distance. Using a sample thickness of 2 mm and a sample centre-detector distance of 800 mm, the peak broadening was estimated to be 2.5×10^{-3} Å^{-1}, which was smaller than variations in HA002 peak width due to crystallite size and therefore ignored.

A map over HA crystallite length and strain based on the Williamson-Hall approximation and the HA002 and HA004 peaks calculated for a cross-section through the sample presented in the main article is shown in Figure S7a-c). A map over the crystallite length calculated by the Scherrer equation without Williamson-Hall analysis is provided in Figure S7d) for comparison.

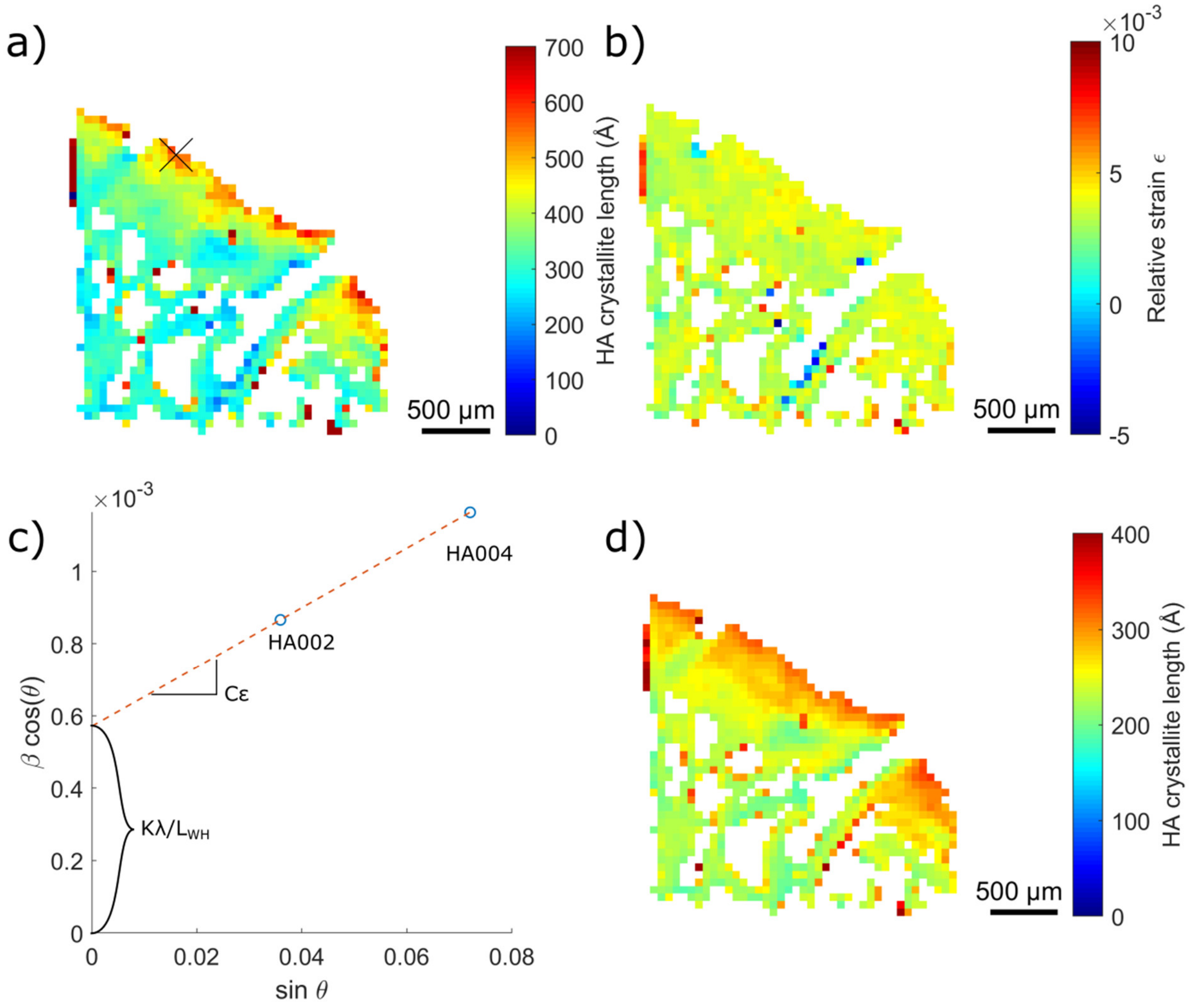

Figure S7 Estimation of HA crystallite length and strain in the same cross-section shown in Figure 2 and 3 in the article. a) Estimate of crystallite lengths L_{WH} using the Williamson-Hall approximation and the measured widths of the HA002 and HA004 peaks. b) Estimated relative strain ϵ in the *00l direction* from the Williamson-Hall approximation. c) Williamson-Hall plot (Equation S7.4) at the point marked with X in a). d) Estimated crystallite length using the Scherrer equation and the HA002 peak.

S8 Correlation between HA orientation and bone morphology

To correlate the HA crystallite *c*-axis orientation obtained by XRDTT with the bone trabecula morphology obtained by PPC-CT, a simple analysis based on the fast Fourier transform (FFT) was done. From the reconstructed PPC-CT volume of the bone sample, and after masking away the cartilage and sample holder regions, the absolute square magnitude $|F|^2$ was calculated. Cross-sections of the absolute square magnitude of the 3D FFT from the PPC-CT data sample volume is shown in Figure S8.1b-d). The high intensity regions in Figure S8.1b-d) take the shape of an ellipsoid. By performing an ellipsoid fit (cf. Figure S8.1e) around the high intensity region centred at the origin in Fourier space ($\mathbf{k}=\mathbf{0}$), the directions of the three semi axes $\mathbf{e}_1$, $\mathbf{e}_2$ and $\mathbf{e}_3$ were found. The axis $\mathbf{e}_3$ coincides approximately with the observed HA *c*-axis orientation, which supports that there is a correlation between the bone trabecula morphology and the HA orientation. Figure S8.1f) shows the HA *c*-axis orientation corresponding to the cross-section in a), which is the same as used in Figure S3a. Figure S8.1 f) gives the angular difference between the ellipsoid $\mathbf{e}_3$ axis and the local orientation vector. The average orientation difference in the slice studied was 29° ± 14°. The reason for this offset could be that the 2D plane chosen for the FFT analysis does not coincide with the principal axes. Additionally, to assess the degree of orientation the 2D FFT and the Hermans' parameter were calculated for the cross-section in Figure S8.1, as shown in Figure S8.2.

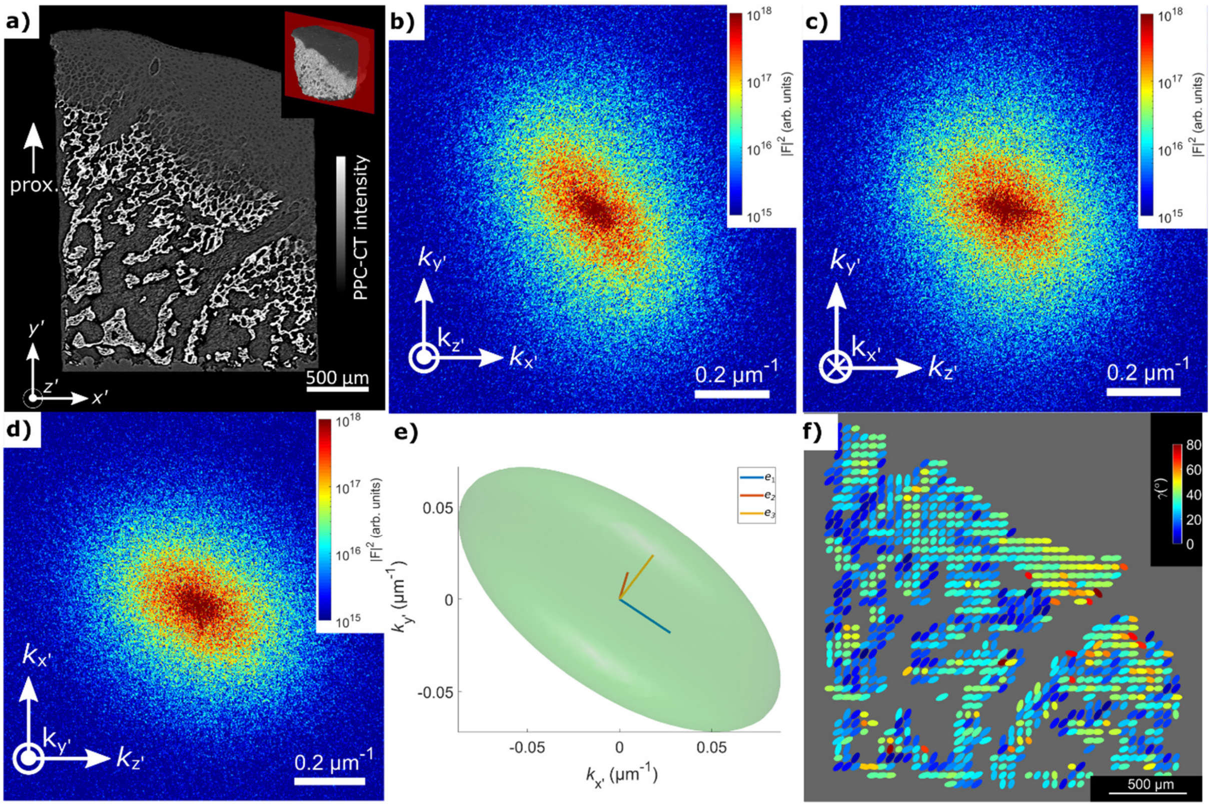

Figure S8.1 Relation between bone trabecula morphology and HA orientation. a) PPC-CT cross-section, identical to the one presented in Figure 2 and 3. b) Absolute magnitude squared of the FFT of the full 3D PPC-CT volume of the sample, sliced in orthogonal cross-sections around the origin in Fourier space ($\mathbf{k}=\mathbf{0}$). e) Ellipsoid fit of the intensities. The principal axes of the ellipsoid have been indicated. f) XRDTT reconstructed corresponding to the cross-section in a). The direction of the oriented ellipsoids indicates the preferred HA *c*-axis orientation. The colours of the ellipsoids indicate the angle between the e_3 axis in e) and the preferred HA *c*-axis orientation, $\gamma(\mathbf{r}')$. All ellipsoids are displayed with the same shape.

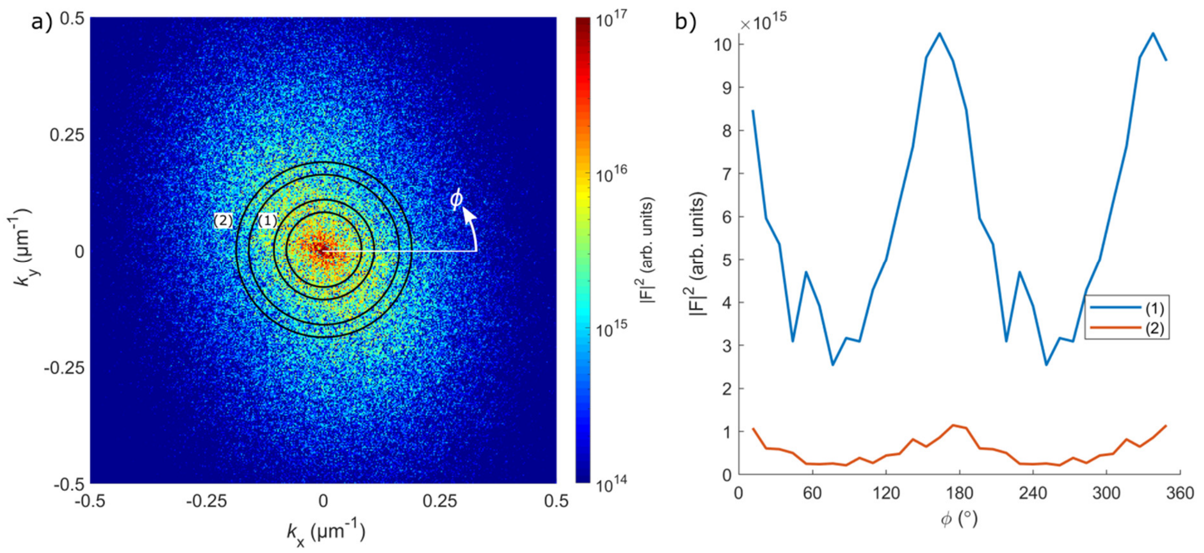

Figure S8.2 Azimuthal intensity variations around the origin (**k**=**0**) in Fourier space from a 2D cross-section. a) 2D magnitude squared of the FFT of the 2D cross-section in Figure S8.1a. The rings indicate selected regions for regions in φ of k = 0.094 μm^{-1} and k = 0.17 μm^{-1}, corresponding to real-space structural features of 67 µm and 35 µm, respectively. b) Azimuthal variations of $|F|^2$ in a). The calculated Hermans' orientation parameter for (1) and (2) are 0.14 and 0.18, respectively.

S9 HA orientation obtained from the trochlea sample

In addition to the results from the femoral medial condylar sample presented in the main text, a second sample was measured, obtained from the lateral trochlear ridge of the same limb of the same individual. The same features in HA orientation were observed. Figure S9 shows the HA orientation in a cross-section through the sample, where the HA *c*-axis orientation tended towards the interface.

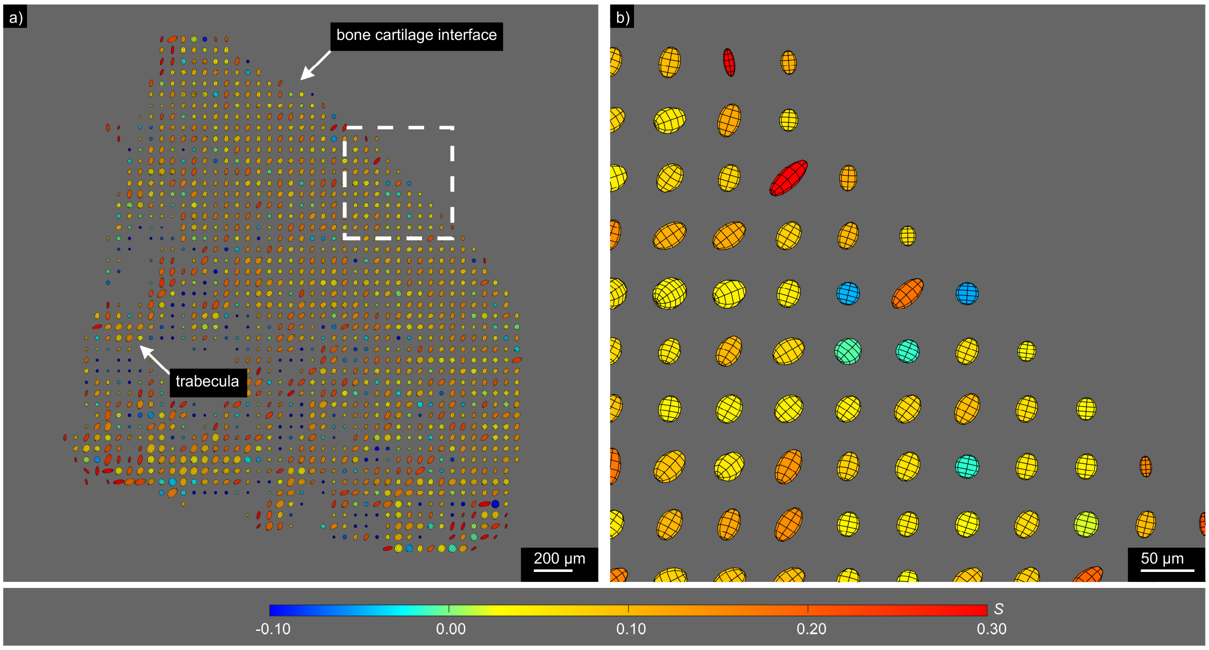

Figure S9 XRDTT cross-section of the second sample studied, obtained from the trochlea of the same limb of the same individual. The ellipsoids indicate preferred orientation of the HA crystallite *c*-axis, tending towards the bone cartilage interface.

References

1. Liebi, M. *et al.* Nanostructure surveys of macroscopic specimens by small-angle scattering tensor tomography. *Nature* **527**, 349–352 (2015).

2. Liebi, M. *et al.* Small-angle X-ray scattering tensor tomography: Model of the three-dimensional reciprocal-space map, reconstruction algorithm and angular sampling requirements. *Acta Crystallogr. Sect. A Found. Adv.* **74**, 12–24 (2018).

3. Als-Nielsen, J. & McMorrow, D. *Elements of modern X-ray physics*. (John Wiley & Sons, 2011).

4. Ashiotis, G. *et al.* The fast azimuthal integration Python library: PyFAI. *J. Appl. Crystallogr.* **48**, 510–519 (2015).

5. Bunk, O. *et al.* Multimodal x-ray scatter imaging. *New J. Phys.* **11**, 123016 (2009).

6. van Aarle, W. *et al.* Fast and flexible X-ray tomography using the ASTRA toolbox. *Opt. Express* **24**, 25129 (2016).

7. van Aarle, W. *et al.* The ASTRA Toolbox: A platform for advanced algorithm development in electron tomography. *Ultramicroscopy* **157**, 35–47 (2015).

8. Kak, Avinash C., Slaney, M. *Principles of Computerized Tomographic Imaging*. (Society for Industrial and Applied Mathematics, 1987).

9. O'Haver, T. *A Pragmatic Introduction to Signal Processing with applications in scientific measurement. University of Maryland at College Park* (2019).

10. Williamson, G. K. & Hall, W. H. X-ray line broadening from filed aluminium and wolfram. *Acta Metall.* **1**, 22–31 (1953).

11. Zsigmondy, R. & Scherrer, P. Bestimmung der inneren Struktur und der Größe von Kolloidteilchen mittels Röntgenstrahlen. in *Kolloidchemie Ein Lehrbuch* 387–409 (1912). doi:10.1007/978-3-662-33915-2_7

12. Turunen, M. J. *et al.* Bone mineral crystal size and organization vary across mature rat bone cortex. *J. Struct. Biol.* **195**, 337–344 (2016).

13. Danilchenko, S. N. *et al.* Determination of the bone mineral crystallite size and lattice strain from diffraction line broadening. *Cryst. Res. Technol.* **37**, 1234–1240 (2002).

14. Beale, A. M., Jacques, S. D. M., Gibson, E. K. & Di Michiel, M. Progress towards five dimensional diffraction imaging of functional materials under process conditions. *Coord. Chem. Rev.* **277–278**, 208–223 (2014).
